# Phylogenetic patterns and the adaptive evolution of osmoregulation in fiddler crabs (Brachyura, *Uca*)

**DOI:** 10.1371/journal.pone.0171870

**Published:** 2017-02-09

**Authors:** Samuel Coelho Faria, Diogo Borges Provete, Carl Leo Thurman, John Campbell McNamara

**Affiliations:** 1 Departamento de Biologia, Faculdade de Filosofia, Ciências e Letras de Ribeirão Preto, Universidade de São Paulo, Ribeirão Preto SP, Brazil; 2 Departamento de Ciências Ambientais, Universidade Federal de São Carlos, Sorocaba SP, Brazil; 3 Gothenburg Global Biodiversity Centre, Göteborg, Sweden; 4 Department of Biology, University of Northern Iowa, Cedar Falls IA, United States of America; 5 Centro de Biologia Marinha, Universidade de São Paulo, São Sebastião SP, Brazil; Xiamen University, CHINA

## Abstract

Salinity is the primary driver of osmoregulatory evolution in decapods, and may have influenced their diversification into different osmotic niches. In semi-terrestrial crabs, hyper-osmoregulatory ability favors sojourns into burrows and dilute media, and provides a safeguard against hemolymph dilution; hypo-osmoregulatory ability underlies emersion capability and a life more removed from water sources. However, most comparative studies have neglected the roles of the phylogenetic and environmental components of inter-specific physiological variation, hindering evaluation of phylogenetic patterns and the adaptive nature of osmoregulatory evolution. Semi-terrestrial fiddler crabs (*Uca*) inhabit fresh to hyper-saline waters, with species from the Americas occupying higher intertidal habitats than Indo-west Pacific species mainly found in the low intertidal zone. Here, we characterize numerous osmoregulatory traits in all ten fiddler crabs found along the Atlantic coast of Brazil, and we employ phylogenetic comparative methods using 24 species to test for: (i) similarities of osmoregulatory ability among closely related species; (ii) salinity as a driver of osmoregulatory evolution; (iii) correlation between salt uptake and secretion; and (iv) adaptive peaks in osmoregulatory ability in the high intertidal American lineages. Our findings reveal that osmoregulation in *Uca* exhibits strong phylogenetic patterns in salt uptake traits. Salinity does not correlate with hyper/hypo-regulatory abilities, but drives hemolymph osmolality at ambient salinities. Osmoregulatory traits have evolved towards three adaptive peaks, revealing a significant contribution of hyper/hypo-regulatory ability in the American clades. Thus, during the evolutionary history of fiddler crabs, salinity has driven some of the osmoregulatory transformations that underpin habitat diversification, although others are apparently constrained phylogenetically.

## Introduction

Habitat salinity is a key element guiding the evolution of osmoregulation in the Decapoda, imposing the premise that natural selection would be the main driving mechanism [1–4]. Indeed, osmoregulatory ability in decapod groups from different habitats is reflected in the salinity of their osmotic niches. To illustrate, fully marine decapods do not maintain a significant osmotic gradient between their hemolymph and the surrounding seawater [3,5]. Such isosmoticity is a consequence of their stable osmotic and ionic environment, elevated body permeability, and limited abilities for salt uptake and secretion. In contrast, freshwater decapods maintain a strong hyperosmotic gradient against their osmotically stable external medium by means of various salt uptake mechanisms [6]. In osmotically capricious environments like coastal lagoons, estuaries and mangroves, salt and water availability varies considerably, and most species can both hyper- and hypo-osmoregulate their hemolymph, *i*. *e*., uptake or secrete salt, respectively [3,7]. Thus, there is an intimate linkage between ambient salinity and osmoregulatory ability in aquatic decapods.

However, the link between osmotic niche and osmoregulation in semi-terrestrial and terrestrial decapods is not as clear-cut as it is in aquatic forms, since aerial exposure constitutes an osmotic challenge similar to concentrated seawater [2,8,9,10]. Terrestrial decapods exhibit strong osmoregulatory capabilities, *i*. *e*., they maintain their hemolymph osmolality in air and across a wide range of salinities, and are particularly able to hypo-osmoregulate their hemolymph osmolality below that of the medium and of the branchial chamber fluid [2,9]. Water lost by transpiration is replaced by ingested water absorbed across the gut, excess salt being excreted by the antennal glands and gills, maintaining body water content [11]. This ability to hyper-osmoregulate on land favors sojourns into terrestrial burrows or into dilute media, and provides a safeguard against hemolymph dilution.

Early comparative studies on semi-terrestrial and terrestrial crabs revealed that osmoregulatory capability, especially the ability to secret salt, underlies emersion and a life more removed from water sources [8,9,12,13]. Such studies employed submerged crabs to evaluate osmoregulatory ability rather than desiccation tolerance, revealing stronger hyper-/hypo-osmoregulatory capabilities in the more terrestrial than aquatic species. Terrestrial adaptation in crabs ranges from intermittent emersion as seen in some potamid and grapsid crabs, to those that do not require regular immersion, their water being obtained from food or dew, as found in species of *Geosesarma* [11]. Thus, osmoregulatory capability and independence from water appear to form a continuous gradient when viewed across species. However, interspecific variation in the osmoregulatory physiology of semi-terrestrial crabs is poorly known, and is particularly scant as regards the adaptive role of habitat salinity when evaluated from a phylogenetic perspective. Most phylogenetic comparative studies are restricted to palaemonid shrimps [6, 14].

*Uca* is a monophyletic genus of semi-terrestrial fiddler crabs that occur in the tropical to sub-tropical zones of the Atlantic, Indian and Pacific oceans, from low to high intertidal habitats where water availability varies from fresh to hyper-saline water. These fiddler crabs exhibit different osmoregulatory capabilities [11] and have originated in the low intertidal zone of the Indo-west Pacific [15]. An “evolutionary progression” [15] is suggested to have led to the occupation of the upper intertidal zone. However, the Indo-west Pacific fiddler crabs form a monophyletic group while the American fiddlers are divided into two clades [16–18]. In this alternative scenario, behavioral, ecological, morphological and physiological traits associated with terrestriality such as deeper, high intertidal burrows, better vision, increased water retention and reduced permeability may have evolved independently [17]. Thus, stronger osmoregulatory ability by the more terrestrial, high intertidal American fiddlers compared to the more marine, low intertidal, Indo-west Pacific *Uca* would be expected.

Surprisingly, however, despite the abundance of fiddler crab species and their biogeography, phylogenetic patterns of osmoregulation and the evolution of osmoregulatory traits towards adaptive peaks in *Uca* have not been investigated using modern phylogenetic comparative methods. Shared ancestry during the evolution of fiddler crabs suggests that ‘species’ are not statistically independent units, thus requiring a phylogenetic framework for comparative analysis, since degrees of freedom are inflated, reducing the analytical power of conventional analyses [19–22]. Further, given the intrinsic correlation between historical patterns of speciation and physiology [23], the origin of interspecific osmoregulatory diversity favors the direct inheritance of most functional traits from shared ancestors, rather than lying with environmental factors.

Here, we characterize the osmoregulatory patterns of all ten species of *Uca* from the Atlantic coast of Brazil and compile literature data for a further 14 species from the Caribbean, Central and North America, and the Indo-west Pacific, enabling a detailed analysis of osmoregulatory evolution in *Uca*. Specifically, we tested for (i) phylogenetic pattern in osmoregulatory ability; (ii) salinity as driver of osmoregulatory evolution; (iii) correlation between salt uptake and salt secretion; and (iv) the presence of adaptive peaks in the osmoregulatory ability of the American lineages.

## Materials and methods

### Crab collection and physiological experiments

We collected approximately 70 adult specimens of each of the ten Brazilian *Uca* species [24], either directly from the substrate surface or from their burrows. Crabs were taken randomly during low tide at each locality between June and November 2009–2010. The populations sampled (11≤N≤35 populations per species) are distributed along the Atlantic coast of Brazil between the states of Amapá (2° 35’ 47.50” N; 50° 50’ 53.99” W) and Santa Catarina (27° 26’ 57.44” S; 48° 31’ 34.82” W). The fiddler crab species collected were *U*. *burgersi* Holthuis, 1967, *U*. *cumulanta* Crane, 1943, *U*. *leptodactyla* Rathbun, 1898, *U*. *maracoani* (Latreille, 1802–1803), *U*. *mordax* (Smith, 1870), *U*. *rapax* (Smith, 1870), *U*. *thayeri* Rathbun, 1900, *U*. *uruguayensis* Nobili, 1901, *U*. *victoriana* Von Hagen, 1987, and *U*. *vocator* (Herbst, 1804).

The range of approximately 25° in latitude sampled embraces an extensive variety of habitat conditions, including populations ranging from fresh (<0.5 ‰S, 18 mOsm/kg H_*2*_O) to hyper-saline water (40 ‰S, 1,203 mOsm/kg H_*2*_O), found at temperatures from 22 to 36°C, in surface sediments including coarse to fine grained sand to mud (see [24–25] for detailed habitat characteristics). Habitat salinities were measured in the closest available water source such as tide pools or streams, and were classified as oligohaline (0.5 to 5 ‰S), mesohaline (5 to 18 ‰S), polyhaline (18 to 30 ‰S) or euhaline (30 to 40 ‰S) [26]. The choice of the closest water source follows previous protocols [27–31], enabling direct comparison. Further, variability in population salinity is incorporated into the mean habitat salinity for each species, diluting occasional outliers into the species’ salinity range, expressed as the mean ± standard error.

The crabs were transported to the Centro de Biologia Marinha, Universidade de São Paulo, in São Sebastião, São Paulo state, in groups of 3–25 specimens each, in plastic containers holding sponges moistened with water from each collecting site. In the laboratory, from 20 to 50 crabs of each species were held in plastic boxes in an artificial medium isosmotic to that from the collection site at a constant temperature of 23°C for 3 days prior to experiments. Crabs were not fed during experiments.

Groups of from three to five, adult, non-ovigerous, intermolt crabs of either sex, of carapace width greater than 8.0 mm, from each population were exposed for 5 days in small plastic bowls containing ≈50 mL of one of 14 experimental media, ranging from distilled water to concentrated seawater (3,550 mOsm/kg H_2_O, 118 ‰S) (30 mOsm/kg H_2_O = 1 ‰S). Crabs were not fed during the experiments. After 2 days, most mechanisms of anisosmotic extracellular regulation become adjusted, maintaining a dynamic equilibrium between salt uptake and secretion [32]. Experimental hypo- and hyper-osmotic media were prepared by dissolving artificial sea salt (Instant Ocean^®^, Blacksburg, VA) in distilled water, pH being adjusted to 8.0 using 0.5 to 3 M HCl or NaOH as required (DM-PV pHmeter, Digimed, SP). Each bowl held a 3–4 mm deep water layer, maintaining the crab’s body emerged while permitting voluntary, periodic gill wetting, thus simulating natural access to water, allowing evaluation of osmoregulatory ability rather than desiccation tolerance. These experiments were conducted in accordance with previous protocols [27–31] enabling direct comparison.

Mortality was recorded every 24 h. A crab was considered ‘dead’ if it could not right itself after being turned upside down. At the end of the 5-day exposure period, a 10-μL hemolymph sample was drawn from the arthrodial membrane between the basis and coxa of the third pereiopod of live crabs using a #27-gauge needle coupled to a chilled 1.0-mL syringe. The osmolality of water samples from the collecting sites, of the experimental media, and of the hemolymph samples was measured in 10-μL aliquots using a vapor pressure osmometer (Wescor 5520 XR, Logan, UT).

### Osmoregulatory traits

Nine indicators of osmoregulatory ability were measured or calculated: the osmolality of (i) water at the collecting site, and of (ii) the hemolymph; (iii) the lower (LL_50_) and (iv) upper (UL_50_) lethal osmotic limits [osmolality of the external medium at which 50% mortality was recorded, calculated using a Probit analysis [33]; (v) isosmotic concentration (IC), obtained from the intersection of the osmoregulatory response curve (generated from the adjustment of a third degree polynomial [*f(x) = a*_*0*_ *+ a*_*1*_*x + a*_*2*_*x*^*2*^ *+ a*_*3*_*x*^*3*^] to the measured hemolymph osmolalities) with the theoretical isosmotic line [f(x) = a_0_ + a_1_x]; and (vi) hemolymph osmolalities at the LL_50_ (Osm_LL50_) and (vii) UL_50_ (Osm_UL50_) also calculated from the osmoregulatory response curves.

Indices of (viii) hyper- (RC_Hyper_) and (ix) hypo-regulatory (RC_Hypo_) capabilities, respectively reflecting the ability to capture or secrete salt were also determined. RC_Hyper_ was calculated as 1 - (IC - Osm_LL50_)/(IC - LL_50_), and RC_Hypo_ as 1 - (Osm_UL50_ - IC)/(UL_50_ - IC). Indices near 1 indicate little change in hemolymph osmolality with variation in external osmolality; values near 0 show that hemolymph osmolality varies substantially with external osmolality, reflecting limited osmoregulatory ability.

The same parameters were calculated or estimated from the literature for an additional 14 species of *Uca* from the Caribbean and North and Central America and the Indo-west Pacific. Specifically, *U*. *arcuata*, *U*. *vocans*, *U*. *lactea* and *U*. *formosensis* were exposed for 14 days (0, 1 and 4 h, 2, 7 and 14 days) to different salinities ranging from 0 to 60 ‰S [34]. For each salinity, the hemolymph osmolality estimated for day 5 was used to generate an osmoregulatory curve adjusted to a third degree polynomial, as performed for the Brazilian *Uca* species. The lower upper and upper critical osmotic limits were considered to be the lowest and highest salinities in which the species were able to survive over the interval from 2 to 7 days. The upper critical osmotic limit of *U*. *inversa* [35] was inferred from the osmoregulatory curve: since this species was exposed up to 50 ‰S, the UL_*50*_ was assumed to be the highest salinity for which hemolymph data were available (45 ‰S). Since no mortality was detected at the lowest salinity, the LL_*50*_ was phylogenetically imputed using the R package Rphylopars [36], employing an Ornstein-Uhlenbeck model.

### Phylogenetic comparative analyses

Comparative analyses were conducted using a Bayesian phylogenetic tree that included 55 species of *Uca* [37], inferred from nucleotide sequences of mitochondrial 16S and cytochrome c oxidase subunit I rDNA, and nuclear 28S rDNA genes. This is the only phylogeny available with actual branch lengths in terms of genetic divergence. The tree was pruned to match the 24 species for which osmoregulatory data were available. *Uca crenulata* was excluded from the analysis as this crab was not sampled in the phylogeny. A description of all comparative analyses performed is available in the R Markdown document (S1 File).

Phylogenetic patterns in the data were evaluated by autocorrelation analyses, and are presented as phylogenetic correlograms for each osmoregulatory trait, employing Moran’s *I* autocorrelation coefficient for four distance classes [38–39]. These classes display variation in trait similarity from lower to higher hierarchical levels, showing phylogenetic signal between pairs of species as a function of phylogenetic distance. Moran’s *I* varies from -1.0 to +1.0; a significant positive value indicates high similarity between closely related species while a significant negative value denotes that related species are dissimilar. The analysis was performed using the Phylogenetic Analysis in Macroecology application (PAM v0.9 beta) [40].

The hypotheses that salinity is a driver of osmoregulatory evolution, and that hyper- and hypo-osmoregulatory abilities might have coevolved, were tested using a Phylogenetic Generalized Least Squares (PGLS) model. This method assumes correlated residual variation among species due to their shared ancestry [41–43] and is appropriate for evaluating cross-species data. The procedure was performed while simultaneously estimating the α parameter of the Ornstein-Uhlenbeck (O-U) evolutionary model [44]. The α parameter measures the selection strength that centers a trait towards an optimum value, limiting its minimum and maximum values [45]. If α = 0, the osmoregulatory covariance among species results from stochastic evolutionary changes, or from natural selection with randomly shifting optima, as modeled by the Brownian Motion evolutionary model [45,46]. The analysis was performed using the R [47] packages *nlme* [48] and *ape* [49], setting the minimum significance level at P = 0.05.

To illustrate these hypotheses and to evaluate their historical context, ancestral states were estimated using a Maximum Likelihood method under Brownian Motion, by re-rooting the tree at each node and calculating independent contrasts, providing the most likely description of the data over the tree [20]. Ancestral states and 95% Confidence Intervals were estimated using the fastAnc function in *phytools* [50]. Ancestral values were visualized using the contMap function [51].

The presence and distribution of adaptive peaks in the nine osmoregulatory traits for the American lineages were tested using SURFACE [52]. This method fits several O-U models with different optima using stepwise Akaike’s Information Criteria (AIC), a heuristic indicator of goodness-of-fit, to identify lineages under distinct selective regimes [52], beginning with a single model for the whole tree and adding further regimes for each branch. Many candidate models are generated since the descendents of each branch may have their own optima. Model performance was compared using AIC values in which the lowest value indicates the best model [53]. All improvements in AIC values were accepted during model fitting [52]. Since only the consensus topology was accessible, uncertainty could not be incorporated into the analysis. The selective regimes are discussed with regard to the complexity of the intertidal habitat, particularly salinity and aerial exposure.

## Results

### Osmoregulatory capability

**Fig 1:**
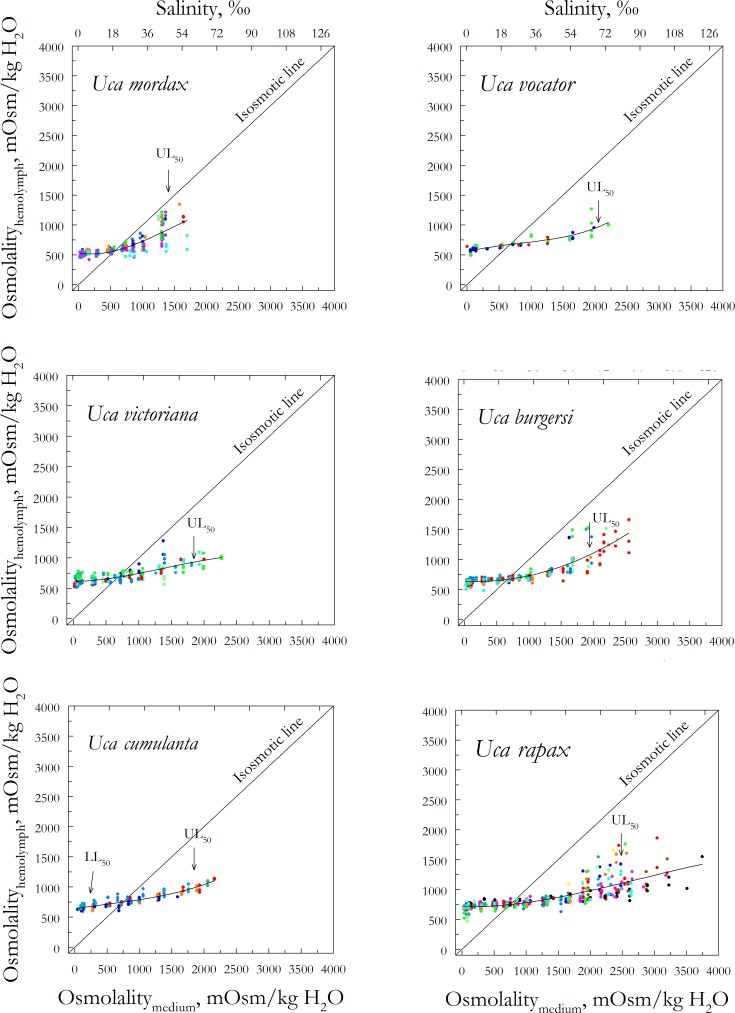
Hemolymph osmoregulatory capability in six *Uca* species. Species are color-coded by population ordered on habitat salinity, after 5-days direct transfer from collecting site water to each different salinity. Populations of each species were collected from dilute (blue) to concentrated (red) media. Data (in mOsm/kg H_2_O) represent single measurements from single individuals in each population (8 ≤ N ≤ 51) and are adjusted to a third degree polynomial equation (0.61 ≤ R^2^ ≤ 0.84). 1 ‰ S = 30 mOsm/kg H_2_O.

**Fig 2:**
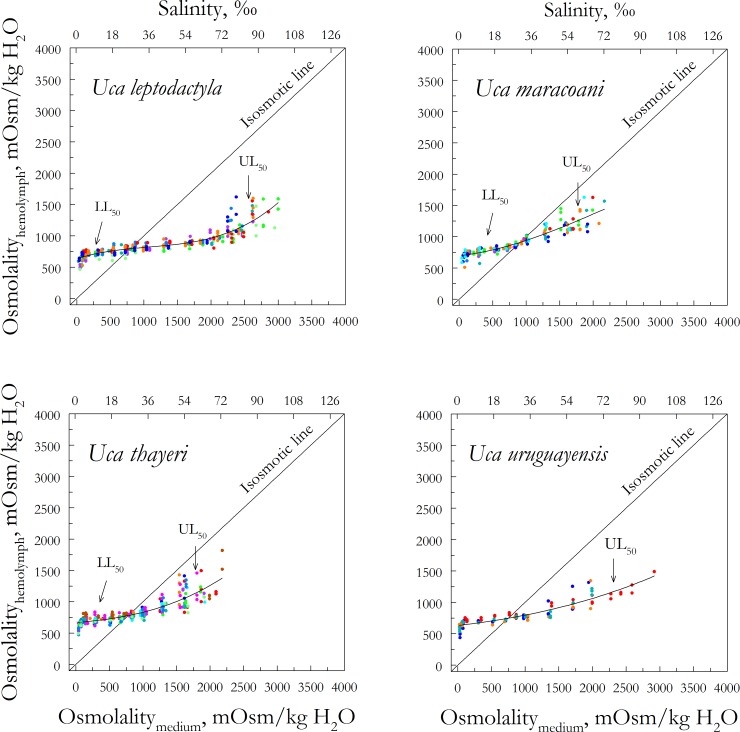
Hemolymph osmoregulatory capability in four *Uca* species. Species are color-coded by population ordered on habitat salinity, after 5-days direct transfer from collecting site water to each different salinity. Populations of each species were collected from dilute (blue) to concentrated (red) media. Data (in mOsm/kg H_2_O) represent single measurements from single individuals in each population (12 ≤ N ≤ 57) and are adjusted to a third degree polynomial equation (0.75 ≤ R^2^ ≤ 0.85). 1 ‰ S = 30 mOsm/kg H_2_O.

The habitat osmolalities of the fiddler crab populations sampled along the Atlantic coast of Brazil varied from nearly fresh water (18 mOsm/kg H_2_O) in some populations of *Uca burgersi*, *U*. *mordax* and *U*. *uruguayensis*, to euhaline water (≈1,200 mOsm/kg H_2_O) in certain populations of *U*. *leptodactyla*, *U*. *rapax*, *U*. *thayeri* and *U*. *uruguayensis* (Table 1, Figs 1 and 2).

**Table 1:** Osmoregulatory traits for all *Uca* species evaluated to date from the American and Indo-west Pacific regions. We provide habitat and hemolymph osmolalities, lower (LL_**50**_) and upper (UL_**50**_) lethal limits, hemolymph osmolalities at LL_**50**_ (Osm_**LL50**_) and UL_**50**_ (Osm_**UL50**_), and isosmotic point (all in mOsm/kg H_**2**_O; 30 mOsm/kg H_**2**_O = 1 ‰ salinity), and calculated indices of hyper- and hypo-regulatory buffering capabilities. Indices close to 1 reflect substantial ability to buffer hemolymph osmolality against variation in external osmolality; values near 0 reveal very limited regulatory ability, reflecting restricted ability to maintain hemolymph osmolality. Species are ordered on increasing habitat salinity within each region.

Species	Habitat osmolality (mOsm/kg H_2_O)	Hemolymph osmolality (mOsm/kg H_2_O)	Lower lethal limit (mOsm/kg H_2_O)	Upper lethal limit (mOsm/kg H_2_O)	Hemolymph osmolality at LL_50_ (mOsm/kg H_2_O)	Hemolymph osmolality at UL_50_ (mOsm/kg H_2_O)	Isosmotic point (mOsm/kg H_2_O)	Hyper-regulatory index	Hypo-regulatory index	Reference
**Americas**										
*U*. *minax*	70	600 ± 25	-	2000	570	1590	667	0.86	0.31	Thurman 2002
*U*. *mordax*	150 ± 38	558 ± 9	-	1453 ± 54	492 ± 9	1057 ± 68	579 ± 15	0.84 ± 0.02	0.43 ± 0.07	Present study
*U*. *vocator*	305 ± 87	653 ± 16	-	2038 ± 105	558 ± 14	967 ± 41	659 ± 24	0.85 ± 0.05	0.78 ± 0.00	Present study
*U*. *victoriana*	338 ± 82	677 ± 27	-	1825 ± 151	602 ± 26	1036 ± 36	676 ± 21	0.89 ± 0.02	0.64 ± 0.09	Present study
*U*. *burgersi*	349 ± 79	670 ± 17	-	1924 ± 63	608 ± 11	1218 ± 77	669 ± 11	0.91 ± 0.02	0.56 ± 0.07	Present study
*U*. *longisignalis*	367	685 ± 15	-	2230	585	1230	693	0.84	0.65	Thurman 2003a
*U*. *cumulanta*	385 ± 107	702 ± 24	151	1905 ± 95	645 ± 10	1006 ± 45	752 ± 26	0.84 ± 0.03	0.79 ± 0.02	Present study
*U*. *spinicarpa*	387 ± 81	644 ± 12	-	2030	600	1130	682	0.88	0.67	Thurman 2003a
*U*. *rapax*	476 ± 63	778 ± 8	-	2475 ± 141	680 ± 14	1195 ± 49	762 ± 12	0.89 ± 0.02	0.74 ± 0.03	Present study
*U*. *uruguayensis*	500 ± 87	759 ± 18	-	2307 ± 185	610 ± 29	1360 ± 51	748 ± 19	0.82 ± 0.02	0.6 ± 0.04	Present study
*U*. *thayeri*	529 ± 63	770 ± 12	99	1783 ± 49	673 ± 15	1153 ± 30	765 ± 11	0.87 ± 0.02	0.61 ± 0.04	Present study
*U*. *panacea*	548 ± 102	796 ± 11	-	2975	660	1440	822	0.75	0.71	Thurman 2003a
*U*. *maracoani*	606 ± 72	835 ± 15	153	1786 ± 92	713 ± 11	1277 ± 44	912 ± 10	0.75 ± 0.01	0.57 ± 0.04	Present study
*U*. *leptodactyla*	609 ± 84	787 ± 11	33	2585 ± 61	620 ± 17	1303 ± 77	800 ± 16	0.77 ± 0.03	0.72 ± 0.04	Present study
*U*. *pugilator*	694 ± 185	850 ± 33	-	3270	620	1660	816	0.76	0.66	Thurman 2003a
*U*. *pugnax*	763 ± 54	805 ± 15	100	2700	700	1600	879	0.77	0.6	Thurman 2003b
*U*. *subcylindrica*	882 ± 599	785 ± 20	100	3200	800	1520	845	0.94	0.71	Thurman 2002
*U*. *crenulata*	917	912 ± 18	58	2910	500	1400	888	0.53	0.75	Thurman 2005
*U*. *major*	1129	997	91	2672	750	1400	930	0.79	0.8	Thurman 2010
*U*. *speciosa*	1149	903	-	2958	700	1400	822	0.85	0.81	Thurman 2010
**Indo-west Pacific**										
*U*. *arcuata*	339 ± 81	715	-	1350	590	1230	906	0.65	0.27	Lin et al. 2002
*U*. *lactea*	1083 ± 24	830	15	1800	500	1700	895	0.55	0.11	Lin et al. 2002
*U*. *vocans*	1179 ± 24	812	15	1800	450	1750	911	0.49	0.06	Lin et al. 2002
*U*. *formosensis*	1257 ± 33	875	15	1800	500	1510	1009	0.49	0.37	Lin et al. 2002
*U*. *inversa*	1260	1177	171	1350	763	1226	1080	0.65	0.46	Spaargaren 1977

*Uca mordax* was the only species found in oligohaline habitats. Mean habitat salinity was 5 ‰S (150 ± 38 mOsm/kg H_2_O) and mean hemolymph osmolality was 558 ± 9 mOsm/kg H_2_O (Table 1, Fig 1). Even so, *U*. *mordax* survived a wide range of experimental salinities, from distilled water to 48 ‰S (1,453 ± 54 mOsm/kg H_2_O) after 5-days exposure. This species’ isosmotic concentration was the lowest (579 ± 15 mOsm/kg H_2_O) of all the species sampled, as was its upper lethal limit. Its hyper-osmoregulatory capacity (0.84 ± 0.02) was strong, but hypo-osmoregulatory ability (0.43 ± 0.07) was moderate (Table 1, Fig 1).

The remaining nine species occupied meso- (5 to 18 ‰S) to polyhaline habitats (18 to 30 ‰S). Mean habitat salinities ranged from 10 ‰S (305 ± 87 mOsm/kg H_2_O) for *U*. *vocator*, to 20 ‰S (609 ± 84 mOsm/kg H_2_O) for *U*. *leptodactyla* (Table 1, Fig 2). *Uca vocator* had the lowest mean hemolymph osmolality (653 ± 16 mOsm/kg H_2_O) and *U*. *maracoani* the highest (835 ± 15 mOsm/kg H_2_O) in their respective habitats (Table 1, Figs 1 and 2). The 5-day lower lethal salinity limit (LL_50_) ranged from less than ≈12 mOsm/kg H_2_O (distilled water) in *U*. *vocator*, *U*. *victoriana*, *U*. *burgersi*, *U*. *rapax* and *U*. *uruguayensis*, to 5 ‰S (≈150 mOsm/kg H_2_O) in *U*. *cumulanta* and *U*. *maracoani*. The 5-day UL_50_ varied from 59 ‰S (1,783 ± 49 mOsm/kg H_2_O) in *U*. *thayeri* to 86 ‰S (2,585 ± 61 mOsm/kg H_2_O) in *U*. *leptodactyla* (Table 1, Fig 2).

The mean osmoregulatory indices for all species considered together were stronger for hyper- (0.84 ± 0.02) than for hypo-osmoregulatory capability (0.68 ± 0.03) (Table 1). The maximum hyper-osmoregulatory index was 0.91 ± 0.02 in *U*. *burgersi*, reaching a minimum of 0.75 ± 0.01 in *U*. *maracoani*. The maximum and minimum hypo-osmoregulatory indices were 0.79 ± 0.02 in *U*. *cumulanta* and 0.56 ± 0.07 in *U*. *burgersi*, respectively (Table 1, Fig 1).

### Phylogenetic pattern

**Fig 3:**
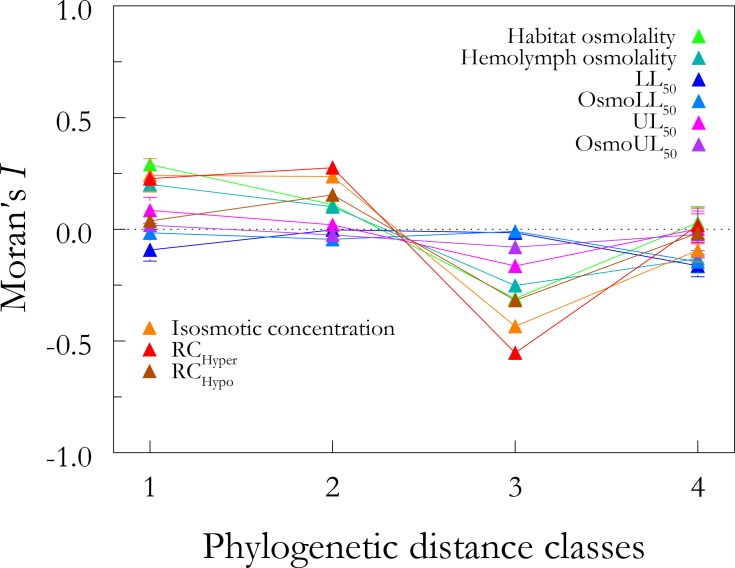
Phylogenetic correlogram showing Moran’s *I* coefficients for 9 osmoregulatory traits at 4 distance classes in the *Uca* phylogeny modified from Shih et al. (2015). Horizontal line at I = 0.0 indicates the expected value under the null hypothesis of no autocorrelation. Habitat and hemolymph osmolalities, isosmotic concentration and hyper-osmoregulatory index (RC_Hyper_) showed positive autocorrelation for the first and second distance classes, shifting to either negative or no autocorrelation for the other two classes, demonstrating strong phylogenetic structuring. However, traits associated with the lower (LL_50_) and upper (UL_50_) lethal limits, and their respective hemolymph osmolalities (Osm_LL50_, Osm_UL50_) and with hypo-osmoregulatory capacity (RC_Hypo_) do not correlate significantly with phylogeny for any distance class.

Habitat and hemolymph osmolalities, isosmotic concentration and hyper-osmoregulatory indices all showed positive autocorrelations for the first and second distance classes, shifting to either negative, or null autocorrelations in the third and fourth distance classes, demonstrating strong phylogenetic structuring (Fig 3). The remaining osmoregulatory parameters (LL_50_ and UL_50_, Osm_LL50_ and Osm_UL50_, and RC_Hypo_) did not correlate with phylogeny, suggesting that these traits were more plastic during the evolution of *Uca* since they differ between pairs of closely related species (Fig 3).

### Trait correlations and ancestral states

**Fig 4:**
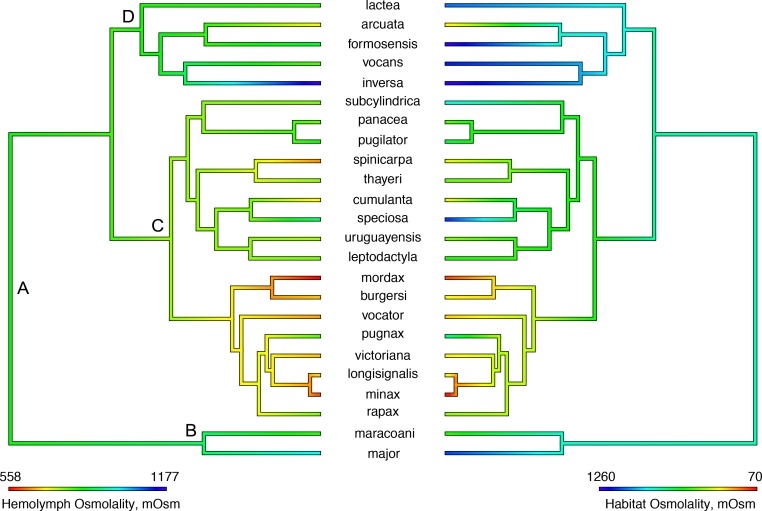
Estimation of ancestral states by maximum likelihood analysis for habitat osmolality (mOsm/kg H_2_O, left panel, 1 ‰ salinity = 30 mOsm/kg H_2_O) and hemolymph osmolality (mOsm/kg H_2_O, right panel) in the fiddler crab topology modified from Shih et al. (2015). Both traits are positively associated (PGLS slope = 0.30, F = 43.8, P < 0.001, α = 2.1) suggesting correlated evolution. A, B, C and D are natural groupings particularly relevant to the interpretation of osmoregulatory evolution [A, genus *Uca*; B and C, American clades; D, Indo-west Pacific clade].

Hemolymph and habitat osmolalities (Fig 4) were correlated (slope = 0.30, F_24,22_ = 43.8, P < 0.001, α = 2.1), suggesting that salinity has driven the evolution of osmoregulatory ability in the crabs’ natural environment. The salinity of the ancestral habitat for *Uca* was estimated to be ≈28 ‰S (828 mOsm/kg H_2_O, 95% CI = 236–1419). Similar values were found for American clade B (29 ‰S, 858 mOsm/kg H_2_O, 423–1293) and the Indo-West Pacific group D (30 ‰S, 898 mOsm/kg H_2_O, 542–1253), although a lower salinity was predicted for the origin of American clade C (21 ‰S, 632 mOsm/kg H_2_O, 333–930; Fig 4, left panel). Thus, hemolymph osmolality is slightly hyper-regulated against the ancestral ambient osmolality at the outset of *Uca* (851 mOsm/kg H_2_O, 647–1056) and for American clades B (901 mOsm/kg H_2_O, 751–1052) and C (768 mOsm/kg H_2_O, 665–871) (Fig 4, right panel). However, at the origin of the Indo-West Pacific clade D, hemolymph osmolality is slightly hypo-regulated (847 mOsm/kg H_2_O, 724–969). This ancestral pattern of hyper- and weak hypo-osmoregulation, suggests a tendency towards isosmoticity at natural habitat salinities (slope = 0.21, F_24,22_ = 24.9, P ≤ 0.001, α = 1.4).

**Fig 5:**
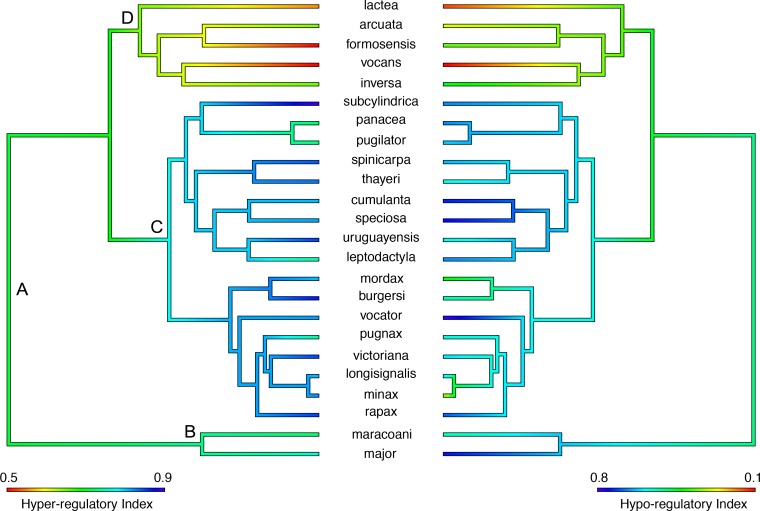
Estimation of ancestral states by maximum likelihood analysis for hyper- and hypo-osmoregulatory indices (left and right panels, respectively) in the fiddler crab topology modified from Shih et al. (2015). Indices close to 1 reflect substantial ability to regulate hemolymph osmolality against variation in external osmolality; values near 0 reveal very limited regulatory ability, reflecting restricted capability to maintain hemolymph osmolality. The evolution of the two indices is not correlated (PGLS slope = 0.11, F = 1.7, P < 0.81, α = 0.6), and neither index is linked to habitat osmolality (PGLS slope ≈0.0, 0.8 ≤ F ≤ 3.3, 0.1 ≤ P ≤ 0.4, 0.3 ≤ α ≤ 1.7). However, an association between the indices is revealed at more inclusive levels. A, B, C and D are natural groupings particularly relevant to the interpretation of osmoregulatory evolution [A, genus *Uca*; B and C, American clades; D, Indo-west Pacific clade].

**Fig 6:**
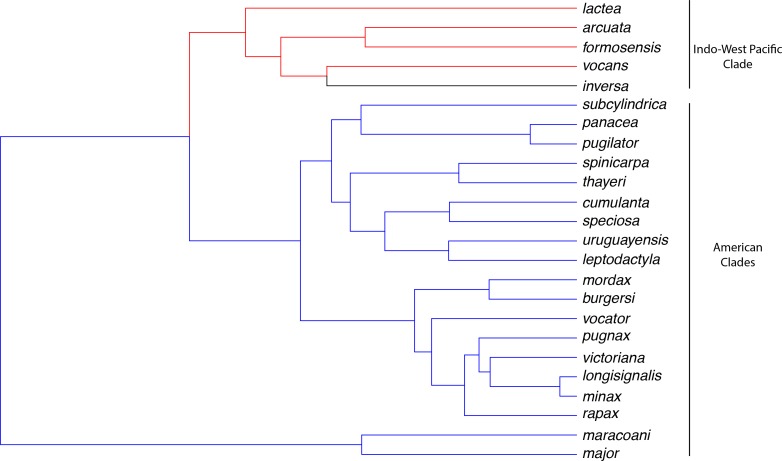
Evolutionary history of shifts in adaptive peaks of *Uca* osmoregulatory ability mapped onto the fiddler crab topology modified from Shih et al. (2015). Each color represents an optimum for all 9 osmoregulatory traits (see Table 1) analyzed together using SURFACE. The first peak (blue) was detected at the tree root and is shared by the American clades, *i*. *e*., the subgenera *Uca*, *Minuca* and *Leptuca*. A second peak (red) occurs in the Indo-west Pacific species, with a shift in *U*. *inversa* (black), the last optimum.

Interestingly, the evolution of hyper- and hypo-osmoregulatory indices (Fig 5) was not correlated (slope = 0.11, F_24,22_ = 1.7, P = 0.18, α = 0.6), suggesting that salt uptake and secretion are not linked. Also, neither capability has evolved with habitat salinity (PGLS slope ≈0.0, 0.8 ≤ F ≤3.3, 0.1 ≤ P ≤ 0.4, 0.3 ≤ α ≤ 1.7). The root states of 0.73 (95% CI = 0.59–0.86) for hyper- and 0.53 (0.13–0.94) for the hypo-osmoregulatory indices (Fig 6) are apparently lower at the origin of the Indo-west Pacific clade [0.65 (0.57–0.74) and 0.38 (0.14–0.62)], but slightly higher at the outset of both American lineages B [0.76 (0.66–0.86) and 0.65 (0.35–0.95)] and C [0.81 (0.74–0.88) and 0.61 (0.40–0.81)], respectively (Fig 5).

### Adaptive landscape of osmoregulatory traits

**Fig 7:**
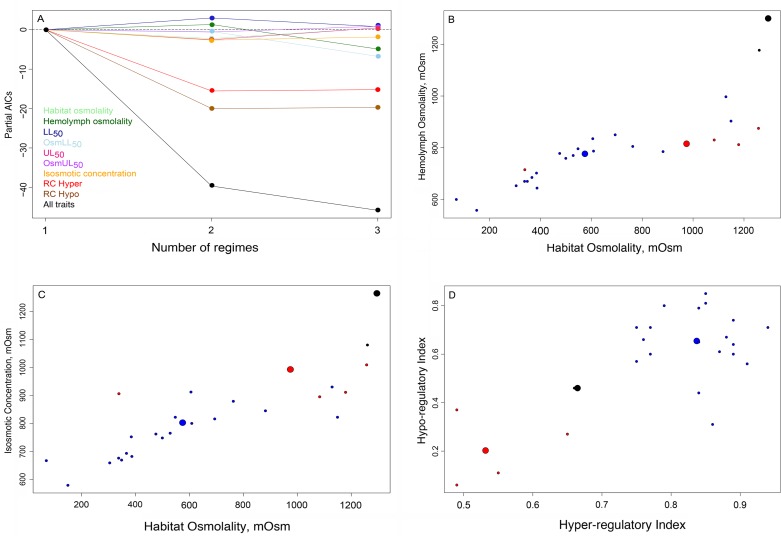
Results of a SURFACE analysis using osmoregulatory traits in 24 *Uca* species. A, Relative contribution of osmoregulatory traits in *Uca* to model fitting, showing the Partial AIC scores for each (colored lines), and for all traits together (multidimensional) (black line). Hyper- (RC_hyper_) and hypo- (RC_hypo_) osmoregulatory indices contributed strongly to model fitting by reducing the AIC values. Hemolymph osmolality, osmolality at LL_50_, and isosmotic concentration contributed moderately to the model; all other osmoregulatory traits reduced model fitting. B, C and D, Estimated position of the species’ traits and adaptive peaks, considering only those traits whose correlations were tested. Large filled circles represent the three optima. Small filled circles are the trait values for each of the 24 fiddler crab species evaluated, colored according to the adaptive peak to which they belong.

When all osmoregulatory traits are considered together, three adaptive peaks were identified (Fig 6). The root optimum is shared by all American clades (blue); the second peak includes the Indo-west Pacific group (red), with a regime shift in *U*. *inversa* (black) (Fig 6). Interestingly, hyper- and hypo-osmoregulatory indices strongly affected model fitting, while hemolymph osmolality, LL_50_, and IC contributed only moderately (Fig 7A).

The distribution of species around each adaptive peak was heterogeneous, particularly for those traits whose correlations were tested (Fig 7B, 7C and 7D). Species were more widely spread around the first optimum, habitat osmolality being the most variable parameter among the American species (Fig 7B and 7C, blue). The osmoregulatory indices tend to be more consistent than the other traits, although the hypo-osmoregulatory index is more variable than the hyper-osmoregulatory index (Fig 7D). The adaptive peaks for the Indo-west Pacific group (Fig 6, red) and for *U*. *inversa* (Fig 6, black) were the most homogeneous (Fig 7B, 7C and 7D, red and black).

## Discussion

This investigation of osmoregulatory ability in *Uca* reveals considerable interspecific variation, a characteristic that poses pertinent questions concerning phylogenetic structuring, adaptive nature, and the transformations that have arisen during the evolutionary history of the group, all addressed here. The ten species from the Atlantic coast of Brazil survive over an extensive experimental salinity range, from 0 to 118 ‰S, displaying just a 0.24-fold alteration in hemolymph osmolality (Δ ≈470 mOsm/kg H_2_O) over a ≈2,000 mOsm/kg H_2_O range of external osmolalities. Traits associated with salt uptake showed a strong phylogenetic pattern at the species level. Salinity appears to be a key factor related to the evolution of the mechanisms underpinning the generation of osmotic gradients. But surprisingly, the ability to regulate hemolymph osmolality does not seem to be linked to salinity. The lesser osmoregulatory ability in the Indo-west Pacific lineage seems to be a consequence of relaxed selection on gill function in the low-intertidal zone. Osmoregulatory ability has evolved towards three adaptive peaks, revealing significant effects of stabilizing selection, especially on hyper- and hypo-osmoregulatory capabilities.

Marked variation in hypo-osmoregulatory ability is present in some species of *Uca* owing to intraspecific variation in hemolymph osmolality in different populations (Figs 1 and 2). This ‘shot-gun effect’ is very clear in *U*. *mordax* in experimental media above 850 mOsm/kg H_2_O, in *U*. *maracoani* above 1,250 mOsm/kg H_2_O, in *U*. *thayeri* and *U*. *burgersi* above 1,500 mOsm/kg H_2_O, in *U*. *rapax* above 1,750 mOsm/kg H_2_O, and in *U*. *leptodactyla* above 2,250 mOsm/kg H_2_O. Wide variation in hemolymph osmolality close to the upper lethal salinity limit is rarely encountered in nature, and reveals reduced hypo-osmoregulatory ability as lethal osmotic challenge is reached. Since only a single salt secreting mechanism based on the basal Na^+^/K^+^/2Cl^-^ symporter and apical Cl^-^ channel has likely evolved in *Uca* [6,7], osmotic stability cannot be maintained against large external gradients. In contrast, hemolymph osmolalities are likely more consistent below isosmoticity since most *Uca* species hyper-osmoregulate their hemolymph at their usual habitat salinities, employing at least two distinct mechanisms of Na^+^ uptake: one based on the apical Na^+^/H^+^ exchanger and apical Na^+^/K^+^/2Cl^-^ symporter, and the other on the apical V(H^+^)-ATPase and Na^+^ channel, both mechanisms sharing an apical Cl^-^/HCO_3_^-^ exchanger [6,7,54].

Positive autocorrelations were detected at the species level for habitat salinity, isosmotic concentration and for those traits associated with salt uptake (hemolymph osmolality and hyper-osmoregulatory index), reflecting the hypo-osmotic media inhabited by most species. Thus, we found that closely related species occupy similar osmotic niches and are physiologically similar, exhibiting similar abilities for salt uptake and equilibrium between water and ion influxes and effluxes. Osmoregulatory ability in the natural osmotic environment tends to be inertial and phylogenetically structured since the customary habitat salinities lie below isosmotic and are routinely encountered by most fiddler crab species. This finding is similar to that for palaemonid shrimps, in which habitat salinity, hemolymph osmolality and osmotic gradient all demonstrate strong phylogenetic signal [6,14]. Conversely, in *Uca*, traits associated with lethal limits and hypo-osmoregulatory capability do not appear to correlate significantly with phylogeny, suggesting that changes in salt secretion ability are not phylogenetically structured. This lower phylogenetic signal seen for osmotic tolerance and salt secretion suggests that the physiological mechanisms that respond to critical osmotic challenge, or conditions not habitually explored by the fiddler crabs, are more plastic than those employed in their natural settings.

Hemolymph osmolality and isosmotic concentration correlate with habitat salinity, suggesting a tendency to reduce the osmotic gradient between the extracellular and ambient media, and salinity as a driver of some osmoregulatory changes [27–29]. In contrast, salinity generally does not correlate with hemolymph osmolality in aquatic decapods; however, it is a key factor in the osmoregulatory evolution of higher taxa, particularly in the palaemonid genera *Macrobrachium* and *Palaemonetes* [6]. Previous studies have proposed that salinity drives evolutionary change in osmoregulatory capacity and in the ultrastructure of ion transporting epithelia, since passive salt and water losses and gains are mitigated by cellular mechanisms that employ various gill and/or renal ion transporters at different cellular locations [6,7,14]. Alterations in the epithelial membrane surface area available for ion transporter insertion, an indicator of ion transport capacity in dilute or concentrated media, have evolved as a function of osmotic gradient in palaemonid shrimps [14].

Most fiddler crabs hyper-osmoregulate their hemolymph osmolality under natural osmotic conditions except for *U*. *major*, *U*. *speciosa* and *U*. *subcylindrica* from the Americas (hemolymph/habitat osmolality ≈895/≈1053 mOsm/kg H_2_O), and *U*. *formosensis*, *U*. *inversa*, *U*. *lactea*, and *U*. *vocans* (≈924/≈1195 mOsm/kg H_2_O) from the Indo-west Pacific that hypo-osmoregulate. Curiously, the indices for hemolymph hyper- and hypo-osmoregulatory ability have not coevolved, so species with stronger capabilities for salt uptake do not necessarily show a greater ability to secrete salt. However, such a tendency is revealed by the increase in both indices at the outset of American clades B and C (Fig 5), and the reduction in both indices at the beginning of the Indo-west Pacific lineage D, compared to the origin of *Uca*. Although salt uptake and secretion capabilities are typical of decapods from environments characterized by fluctuating salinity (*e*. *g*. coastal lagoons and estuaries) [11], salinity does not appear to drive the evolution of either ability in *Uca*.

Shifts in the osmoregulatory capability of *Uca* may result from a trade-off between the number of anterior respiratory and posterior osmoregulatory gills [56]. In grapsid and ocypodid crabs, anterior gill number and surface area decrease while posterior gill surface area increases in those more terrestrial representatives that have developed efficient air-breathing organs, reducing water loss and favoring gas exchange [55–58]. This paradigm in terrestrial crab physiology, extended here to include the American *Uca* species that are morphologically, ecologically and behaviorally more terrestrial than the Indo-West Pacific fiddlers [16–18] is reinforced by the lack of correlation between salinity and osmoregulatory ability. Suggestively, the evolution of osmoregulation in *Uca* is constrained by tolerance to desiccation, the reduction in osmoregulatory capability of the Indo-west Pacific species likely deriving from relaxed selection pressure on gill function in the low intertidal zone.

Stabilizing selection has shaped the evolution of osmoregulation towards three distinct adaptive peaks (Fig 6), rather than the two hypothesized for the American lineages. However, the contribution of each osmoregulatory trait to model fitting differs. The hyper- and hypo-osmoregulatory indices contribute strongly to all adaptive peaks, since they reflect the physiological ability to deal with fluctuating salinities in the intertidal zone. Hemolymph osmolality, osmolality at the lower lethal limit and isosmotic concentration contributed only moderately to the physiological optima. These traits are respectively associated with osmoregulation under natural salinity conditions, exposure to hypo-osmotic media, and with the equilibrium between ion and water fluxes. The other traits, *i*. *e*., the upper lethal limit and osmolality at UL_50_ (Osm_UL50_), reflect conditions not usually encountered in nature, which may justify their minor contribution to model fitting.

The distribution of species traits around each adaptive peak was heterogeneous, particularly in the American clades for habitat and hemolymph osmolalities, and isosmotic concentration. The osmoregulatory indices tend to be more consistent than the other traits, although the hyper-osmoregulatory indices are more variable among the Indo-west Pacific species, while the hypo-osmoregulatory indices are more variable among the American species. Additionally, *U*. *inversa* exhibits an adaptive pattern typical of a hyper-saline environment, usually above 42 ‰S [35], with osmoregulatory traits like elevated hemolymph osmolality and isosmotic concentration lying close to the optimum.

In summary, salinity is a driver of osmoregulatory evolution in semi-terrestrial decapods, at both the species and higher hierarchical levels. Thus, natural selection appears to be the main evolutionary process underpinning some of the physiological transformations that have taken place in *Uca*, creating the functional basis for habitat diversification. Significant effects of phylogenetic relationships are also evident, disclosing an evolutionary landscape still obscure in decapod physiology.

## Supporting information

S1 File R Markdown dynamic document. This file describes all comparative analyses performed in the investigation.
(PDF)
