# Supplementary material for: Phylogenetic patterns and the adaptive evolution of osmoregulation in fiddler crabs (Brachyura, Uca)
Source: PLoS One. 2017 Feb 9;12(2):e0171870. doi: 10.1371/journal.pone.0171870 (PMC5300755; doi:10.1371/journal.pone.0171870)

Analysis from the paper "Phylogenetic patterns and the adaptive **evolution** of osmoregulation in fiddler crabs (Brachyura, *Uca*)"

Samuel Faria, Diogo B. Provete, Carl Thurman, John McNamara

11 january 2017

Contents

Data input and handling **2**

Running SURFACE **6**

Ancestral state estimation using ML **10**
ACE with BM 19

PGLS analyses **27**

```
R.Version()$version.string; R.Version()$platform
```

```
## [1] "R version 3.3.2 (2016-10-31)"
## [1] "x86_64-apple-darwin13.4.0"
```

```
library(surface)
packageVersion("surface")
```

```
## [1] '0.4.1'
```

```
library(phytools)
packageVersion("phytools")
```

```
## [1] '0.5.64'
```

```
library(picante)
packageVersion("picante")
```

```
## [1] '1.6.2'
```

```
library(nlme)
packageVersion("nlme")
```

```
## [1] '3.1.128'
```

```
library(Rphylopars)
packageVersion("Rphylopars")
```

```
## [1] '0.2.9'
```

```
set.seed(1001)#setting the seed to allow reproducibility, since methods below use randomization techniq
```

Data input and handling

The first step is to input the data necessary to run the analysis. Recently, Shih et al. 2015 Zootaxa published a Bayesian analysis for the subgenus Petruca, using the genes 16S rDNA, cytochrome oxidase subunit I genes (COI), and 28S rDNA. This is an improvement over the previously available Maximum Parsimony phylogeny from Rosenberg (2001), which sampled 88 species out of the 102 currently recognized and analysed 236 morphological characters. We had access only to the consensus topology of the Bayesian Inference.

Phylogeny input

```
prune<-read.table("prune_spec.txt")#list of species to which we have trait data
phy<-read.nexus("4_UPM08P_10M_1kbr_-21252.85.tre")#Shih et al. Bayesuan tree
phylog<-prune.sample(t(prune),phy)#prunning the tree so we have only species to which we have trait dat
is.ultrametric(phylog)
```

```
## [1] FALSE
```

```
plot.phylo(phylog);axisPhylo()#plotting pruned phylogeny
```

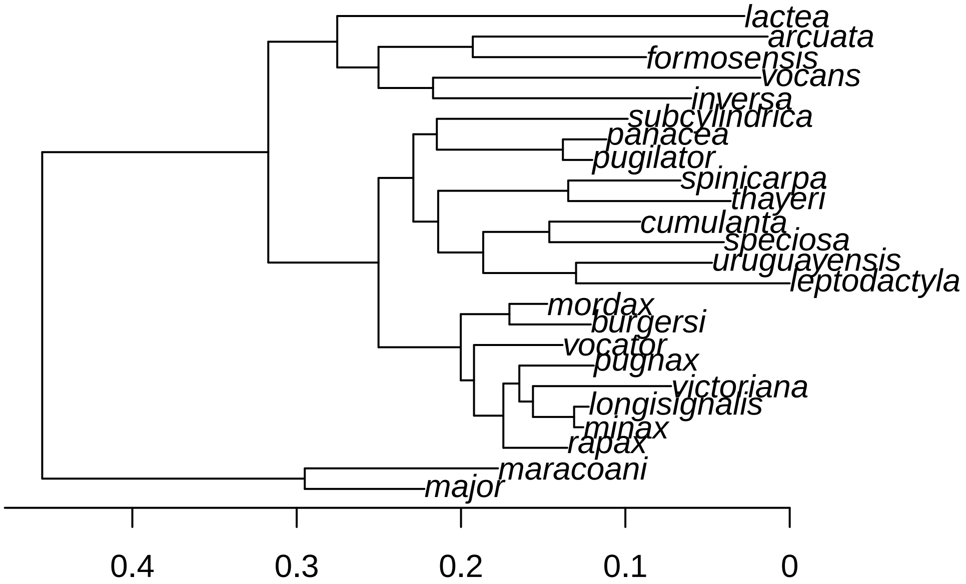


```
phylog<-chronos(phylog)#using the correlated rate model to transform the mean number of substitution pe
```

```
##
## Setting initial dates...
## Fitting in progress... get a first set of estimates
##          Penalised log-lik = -9.012127
## Optimising rates... dates... -9.012127
##
## Done.
```

```
class(phylog) <- "phylo"
plot.phylo(phylog, cex=0.9);axisPhylo()#plotting ultrametric phylogeny
```

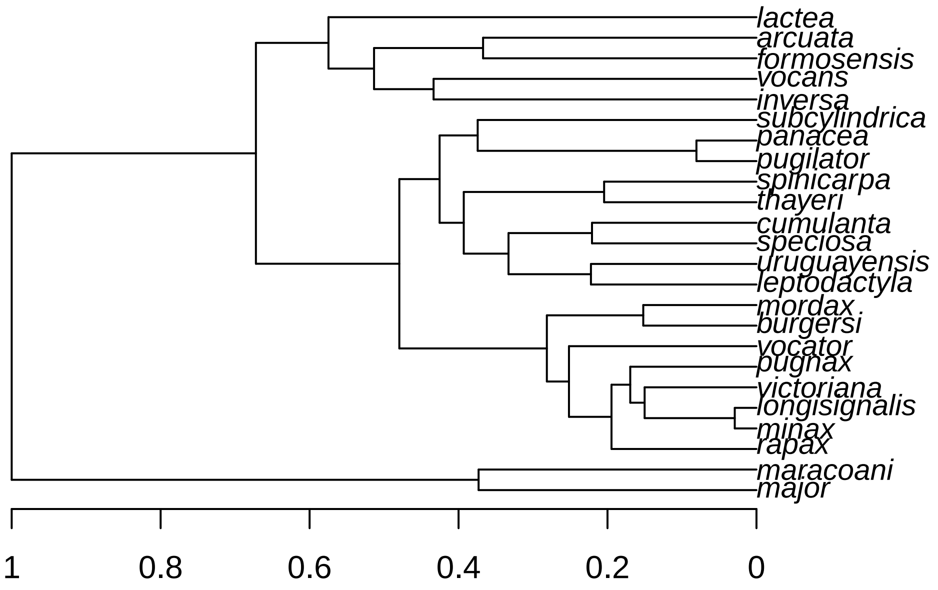


```r
is.ultrametric(phylog)
```

```
## [1] TRUE
```

Trait data input

```r
Trait<-read.table("uca_matrix.txt", h=TRUE)#trait matrix
Traitp<-read.table("uca_matrix_pars.txt", h=TRUE)#data for phylopars
uca.data<-Trait[, c(1:9)]
uca.data
```

```
##               Habitat OsmoHem LLI OsmoLLI  LLS OsmoLLS   PI Hyper Hypo
## thayeri           529     770  99     673 1783    1153  765  0.87 0.61
## vocator           305     653   0     558 2038     967  659  0.85 0.85
## rapax             476     778   0     680 2475    1195  762  0.89 0.74
## longisignalis     367     685   0     585 2230    1230  693  0.84 0.65
## burgersi          349     670   0     608 1924    1218  669  0.91 0.56
## mordax            150     558   0     492 1453    1057  579  0.84 0.44
## pugnax            763     805 100     700 2700    1600  879  0.77 0.60
## panacea           548     796   0     660 2975    1440  822  0.75 0.71
## pugilator         694     850   0     620 3270    1660  816  0.76 0.66
## subcylindrica     882     785 100     800 3200    1520  845  0.94 0.71
## minax              70     600   0     570 2000    1590  667  0.86 0.31
## uruguayensis      500     759   0     610 2307    1360  748  0.89 0.60
## speciosa         1149     903   0     700 2958    1220  822  0.85 0.81
## spinicarpa        387     644   0     600 2030    1130  682  0.88 0.67
## cumulanta         385     702 151     645 1905    1006  752  0.84 0.79
## leptodactyla      609     787  33     620 2585    1303  800  0.77 0.71
## victoriana        338     670   0     602 1825    1036  676  0.89 0.64
## lactea           1083     830  15     500 1800    1700  895  0.55 0.11
## inversa          1260    1177  NA     763 1350    1226 1080  0.65 0.46
## vocans           1179     812  15     450 1800    1750  911  0.49 0.06
## arcuata           339     715   0     590 1350    1230  906  0.65 0.27
## formosensis      1257     875  15     500 1800    1510 1009  0.49 0.37
## maracoani         606     835 153     713 1786    1277  912  0.75 0.57
```

```
## major              1129      997  91      750 2672     1280   930   0.79 0.80
```

```r
p_OU <- phylopars(Traitp, phylog, model = "OU")
imput<-p_OU$anc_recon[1:20,] # Data with imputed species means
p_OU$anc_var[1:20,] # Variances for each estimate
```

```
##                 Habitat OsmoHem      LLI OsmoLLI LLS OsmoLLS PI Hyper Hypo
## rapax                 0       0    0.000       0   0       0  0     0    0
## minax                 0       0    0.000       0   0       0  0     0    0
## longisignalis         0       0    0.000       0   0       0  0     0    0
## victoriana            0       0    0.000       0   0       0  0     0    0
## pugnax                0       0    0.000       0   0       0  0     0    0
## burgersi              0       0    0.000       0   0       0  0     0    0
## mordax                0       0    0.000       0   0       0  0     0    0
## vocator               0       0    0.000       0   0       0  0     0    0
## leptodactyla          0       0    0.000       0   0       0  0     0    0
## uruguayensis          0       0    0.000       0   0       0  0     0    0
## speciosa              0       0    0.000       0   0       0  0     0    0
## cumulanta             0       0    0.000       0   0       0  0     0    0
## thayeri               0       0    0.000       0   0       0  0     0    0
## spinicarpa            0       0    0.000       0   0       0  0     0    0
## pugilator             0       0    0.000       0   0       0  0     0    0
## panacea               0       0    0.000       0   0       0  0     0    0
## subcylindrica         0       0    0.000       0   0       0  0     0    0
## inversa               0       0 1159.489       0   0       0  0     0    0
## vocans                0       0    0.000       0   0       0  0     0    0
## formosensis           0       0    0.000       0   0       0  0     0    0
##                 Clade Hedgpeth
## rapax               0        0
## minax               0        0
## longisignalis       0        0
## victoriana          0        0
## pugnax              0        0
## burgersi            0        0
## mordax              0        0
## vocator             0        0
## leptodactyla        0        0
## uruguayensis        0        0
## speciosa            0        0
## cumulanta           0        0
## thayeri             0        0
## spinicarpa          0        0
## pugilator           0        0
## panacea             0        0
## subcylindrica       0        0
## inversa             0        0
## vocans              0        0
## formosensis         0        0
```

```r
p_OU$anc_recon[1:20,] - sqrt(p_OU$anc_var[1:20,])*1.96 # Lower 95% CI
```

```
##                 Habitat OsmoHem      LLI OsmoLLI  LLS OsmoLLS   PI Hyper
## rapax               476     778   0.0000     680 2475    1195  762  0.89
## minax                70     600   0.0000     570 2000    1590  667  0.86
## longisignalis       367     685   0.0000     585 2230    1230  693  0.84
```

```
## victoriana        338     670   0.0000     602 1825     1036  676  0.89
## pugnax            763     805 100.0000     700 2700     1600  879  0.77
## burgersi          349     670   0.0000     608 1924     1218  669  0.91
## mordax            150     558   0.0000     492 1453     1057  579  0.84
## vocator           305     653   0.0000     558 2038      967  659  0.85
## leptodactyla      609     787  33.0000     620 2585     1303  800  0.77
## uruguayensis      500     759   0.0000     610 2307     1360  748  0.89
## speciosa         1149     903   0.0000     700 2958     1220  822  0.85
## cumulanta         385     702 151.0000     645 1905     1006  752  0.84
## thayeri           529     770  99.0000     673 1783     1153  765  0.87
## spinicarpa        387     644   0.0000     600 2030     1130  682  0.88
## pugilator         694     850   0.0000     620 3270     1660  816  0.76
## panacea           548     796   0.0000     660 2975     1440  822  0.75
## subcylindrica     882     785 100.0000     800 3200     1520  845  0.94
## inversa          1260    1177 122.1233     763 1350     1226 1080  0.65
## vocans           1179     812  15.0000     450 1800     1750  911  0.49
## formosensis      1257     875  15.0000     500 1800     1510 1009  0.49
##               Hypo Clade Hedgpeth
## rapax         0.74     1        2
## minax         0.31     1        1
## longisignalis 0.65     1        2
## victoriana    0.64     1        3
## pugnax        0.60     1        3
## burgersi      0.56     1        2
## mordax        0.44     1        1
## vocator       0.85     1        1
## leptodactyla  0.71     1        2
## uruguayensis  0.60     1        2
## speciosa      0.81     1        4
## cumulanta     0.79     1        2
## thayeri       0.61     1        2
## spinicarpa    0.67     1        2
## pugilator     0.66     1        3
## panacea       0.71     1        2
## subcylindrica 0.71     1        3
## inversa       0.46     2        4
## vocans        0.06     2        4
## formosensis   0.37     2        4
```

```
p_OU$anc_recon[1:20,] + sqrt(p_OU$anc_var[1:20,])*1.96 # Upper 95% CI
```

```
##               Habitat OsmoHem      LLI OsmoLLI  LLS OsmoLLS   PI Hyper
## rapax             476     778   0.0000     680 2475     1195  762  0.89
## minax              70     600   0.0000     570 2000     1590  667  0.86
## longisignalis     367     685   0.0000     585 2230     1230  693  0.84
## victoriana        338     670   0.0000     602 1825     1036  676  0.89
## pugnax            763     805 100.0000     700 2700     1600  879  0.77
## burgersi          349     670   0.0000     608 1924     1218  669  0.91
## mordax            150     558   0.0000     492 1453     1057  579  0.84
## vocator           305     653   0.0000     558 2038      967  659  0.85
## leptodactyla      609     787  33.0000     620 2585     1303  800  0.77
## uruguayensis      500     759   0.0000     610 2307     1360  748  0.89
## speciosa         1149     903   0.0000     700 2958     1220  822  0.85
## cumulanta         385     702 151.0000     645 1905     1006  752  0.84
## thayeri           529     770  99.0000     673 1783     1153  765  0.87
```

```
## spinicarpa        387     644   0.0000     600 2030    1130  682  0.88
## pugilator         694     850   0.0000     620 3270    1660  816  0.76
## panacea           548     796   0.0000     660 2975    1440  822  0.75
## subcylindrica     882     785 100.0000     800 3200    1520  845  0.94
## inversa          1260    1177 255.6043     763 1350    1226 1080  0.65
## vocans           1179     812  15.0000     450 1800    1750  911  0.49
## formosensis      1257     875  15.0000     500 1800    1510 1009  0.49
##                Hypo Clade Hedgpeth
## rapax          0.74     1        2
## minax          0.31     1        1
## longisignalis 0.65     1        2
## victoriana    0.64     1        3
## pugnax        0.60     1        3
## burgersi      0.56     1        2
## mordax        0.44     1        1
## vocator       0.85     1        1
## leptodactyla  0.71     1        2
## uruguayensis  0.60     1        2
## speciosa      0.81     1        4
## cumulanta     0.79     1        2
## thayeri       0.61     1        2
## spinicarpa    0.67     1        2
## pugilator     0.66     1        3
## panacea       0.71     1        2
## subcylindrica 0.71     1        3
## inversa       0.46     2        4
## vocans        0.06     2        4
## formosensis   0.37     2        4
```

```
uca.data$LLI[[19]]<-imput[18,3]
```

Preparing data for ACE and PGLS

```
Habitat=as.matrix(uca.data)[,1]
names(Habitat)=rownames(uca.data)
OsmoHem=as.matrix(uca.data)[,2]
names(OsmoHem)=rownames(uca.data)
PI=as.matrix(uca.data)[,7]
names(PI)=rownames(uca.data)
Hyper=as.matrix(uca.data)[,8]
names(Hyper)=rownames(uca.data)
Hypo=as.matrix(uca.data)[,9]
names(Hypo)=rownames(uca.data)
```

Running SURFACE

Now we'll run Ingram and Mahler's SURFACE. We also set the dAIC (to choose the best model) to either have a difference of at least two units or zero to see if that changes the results. All traits in our database are somehow related, since they are all measures of physiological performance. Therefore, we'll use all traits to run the analysis, because the more traits, the better the chance to find the correct adaptive peakes, as pointed out in the original paper.

```r
uca.phylog<-nameNodes(phylog)
fwd<-runSurface(uca.phylog, uca.data)
rsum<-surfaceSummary(fwd$bwd)
rsum$n_regimes#measures of the regime structure in the final model
```

```
##               k         kprime          deltak                c     kprime_conv
##               3              3               0                0               0
## kprime_nonconv
##               3
```

```r
rsum$alpha#estimate of alpha (selection strength) for each trait
```

```
##    Habitat     OsmoHem         LLI     OsmoLLI         LLS     OsmoLLS
##   5.717441    3.299526    3.927719    3.264251    3.820549 399.068037
##         PI       Hyper        Hypo
##   1.886596    4.702913   72.488003
```

```r
#in the final model
rsum$sigma_squared#estimate of sigma_squared (trait evolution rate)
```

```
##      Habitat      OsmoHem          LLI      OsmoLLI          LLS
## 9.772285e+05 6.188685e+04 1.906690e+04 2.926510e+04 1.592073e+06
##      OsmoLLS           PI        Hyper         Hypo
## 3.116227e+07 2.870286e+04 3.022628e-02 2.230338e+00
```

```r
#for each trait in the final model
rsum$theta#matrix of estimated optima (one per regime per trait)
```

```
##     Habitat   OsmoHem        LLI  OsmoLLI      LLS  OsmoLLS        PI
## a  575.0803  776.5230  49.582101 659.0326 2341.729 1275.895  802.6517
## b  974.4172  815.4186   9.091266 491.7792 1634.807 1547.500  992.7259
## c 1294.9643 1300.0094 226.126326 829.1150 1213.074 1226.000 1264.5193
##       Hyper      Hypo
## a 0.8370528 0.6544752
## b 0.5315377 0.2025000
## c 0.6531692 0.4600000
```

```r
#in the final model
newkk<-length(fwd$bwd)
surfaceTreePlot(uca.phylog, fwd$bwd[[newkk]], cex=1.2, cols = c("blue","red", "black"))
```

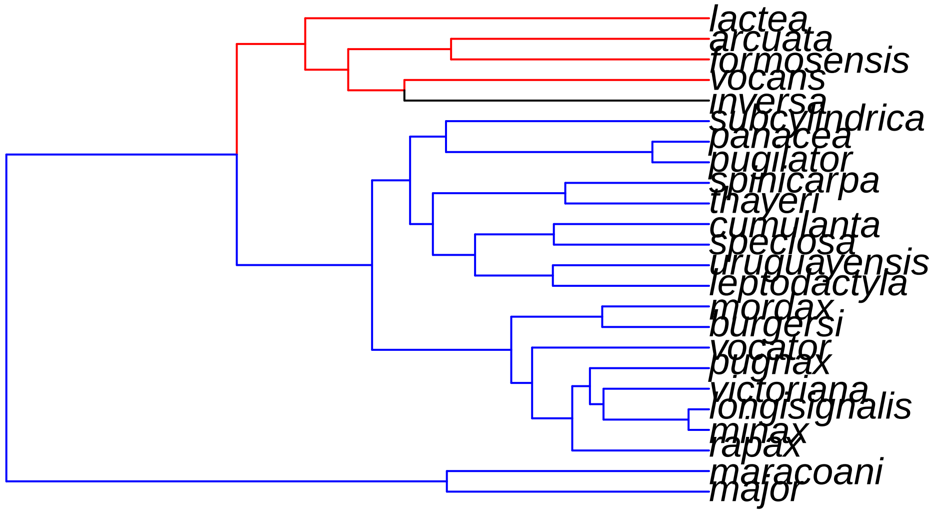

```
surfaceTraitPlot(uca.data, fwd$bwd[[newkk]], cols = c("blue","red", "black"))#for Habitat and OsmoHem
```

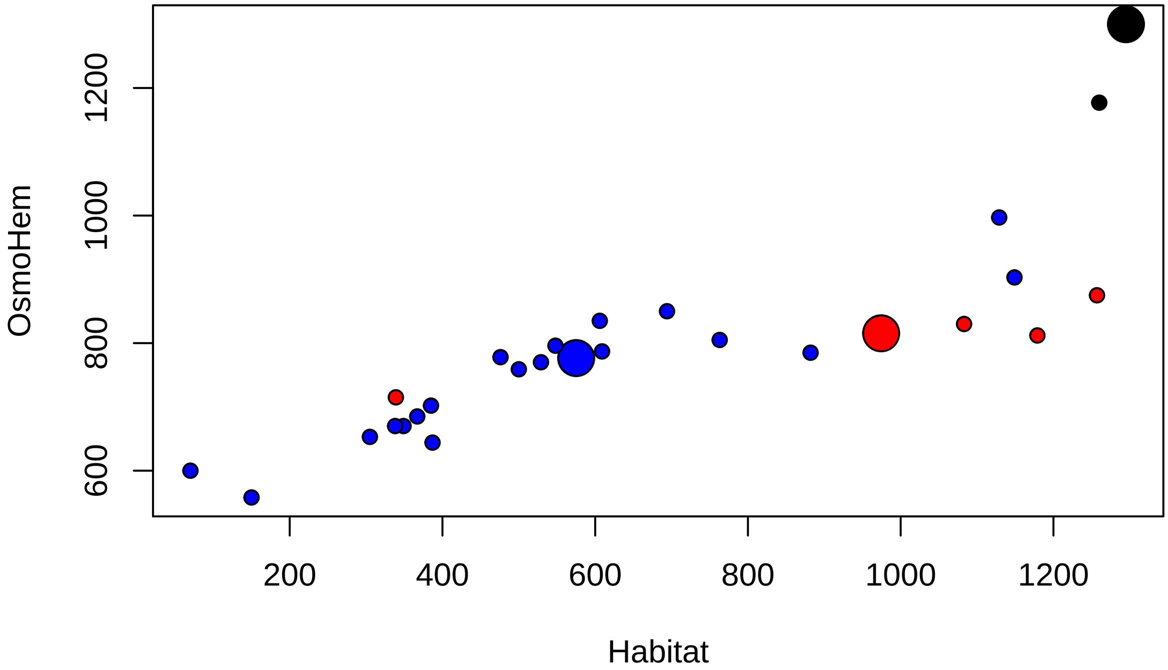


```
surfaceTraitPlot(uca.data, fwd$bwd[[newkk]], c(8,9), cols = c("blue","red", "black"))#for Hyper and Hyp
```

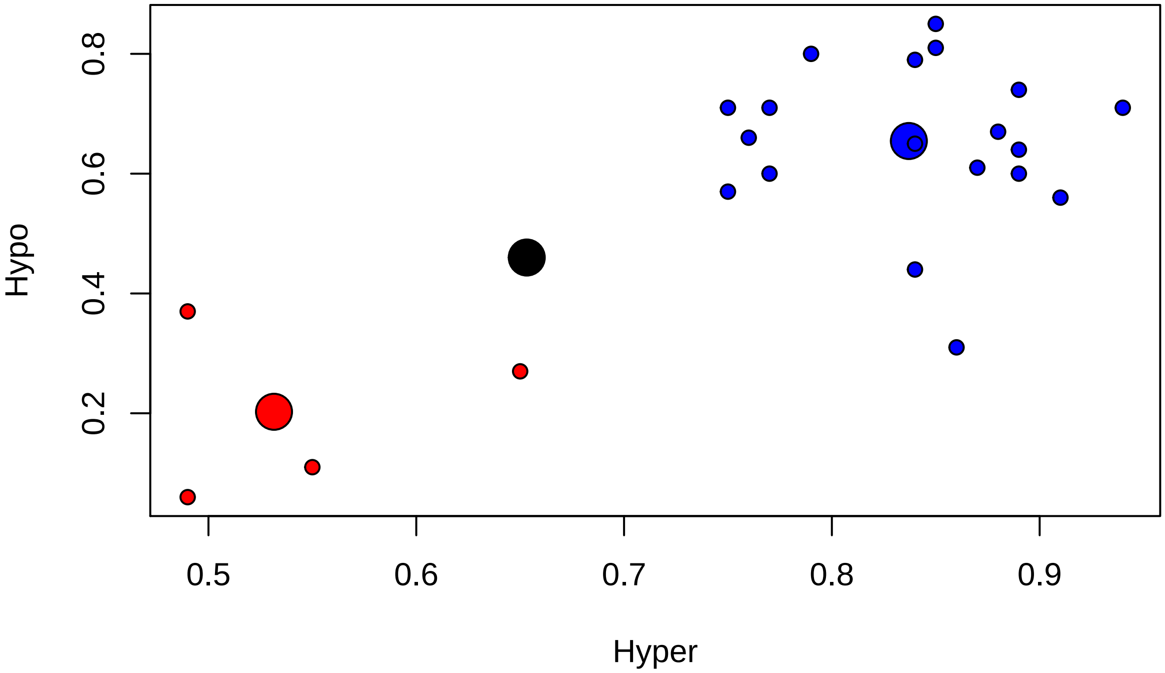


```
surfaceTraitPlot(uca.data, fwd$bwd[[newkk]], c(1,7), cols = c("blue","red", "black"))# for Habitat and
```

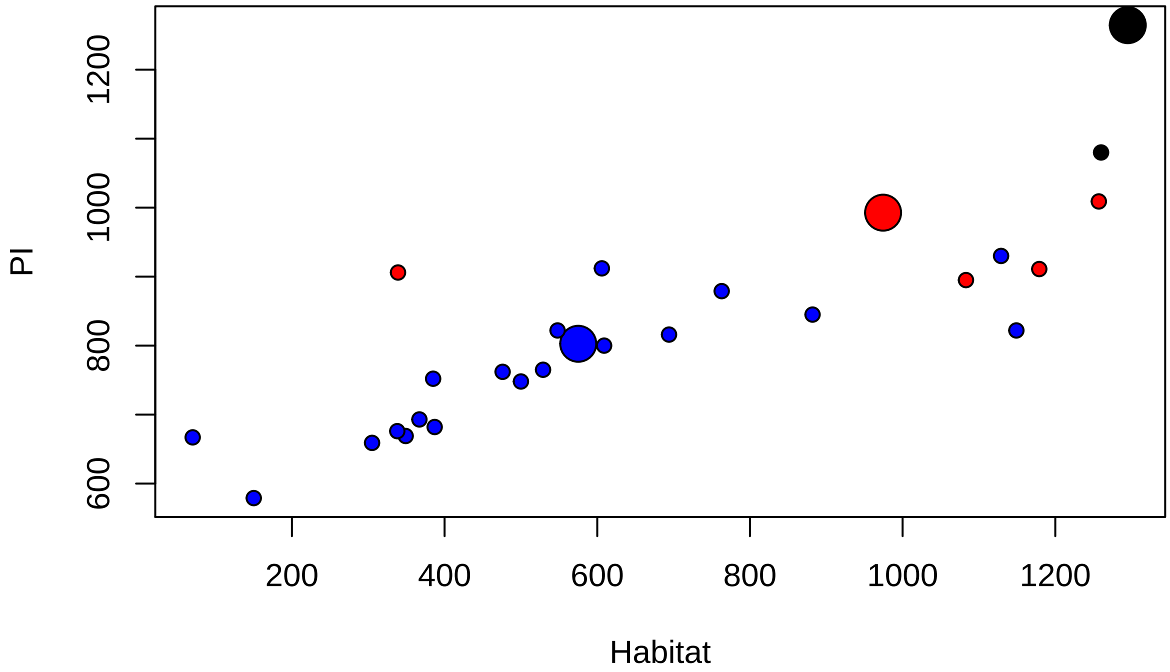

There is an adaptive peak to the clade composed of vocans, formoensis, arcuata, and lactea (red), but a separate one for inversa (black). There is another peak for the American clade (in blue), which is the same peak of the root state.

```
surfaceAICPlot(out = fwd, traitplot = "aic", cols = c(Habitat= "light green", OsmoHem = "dark green", LL
trait_color=c("light green", "dark green", "dark blue", "light blue", "deeppink2", "darkorchid1", "orang
legend("bottomleft", legend = c("Habitat osmol", "Hemolymph osmol", "LL50" , "OsmoLL50", "UL50" , "Osmo
```

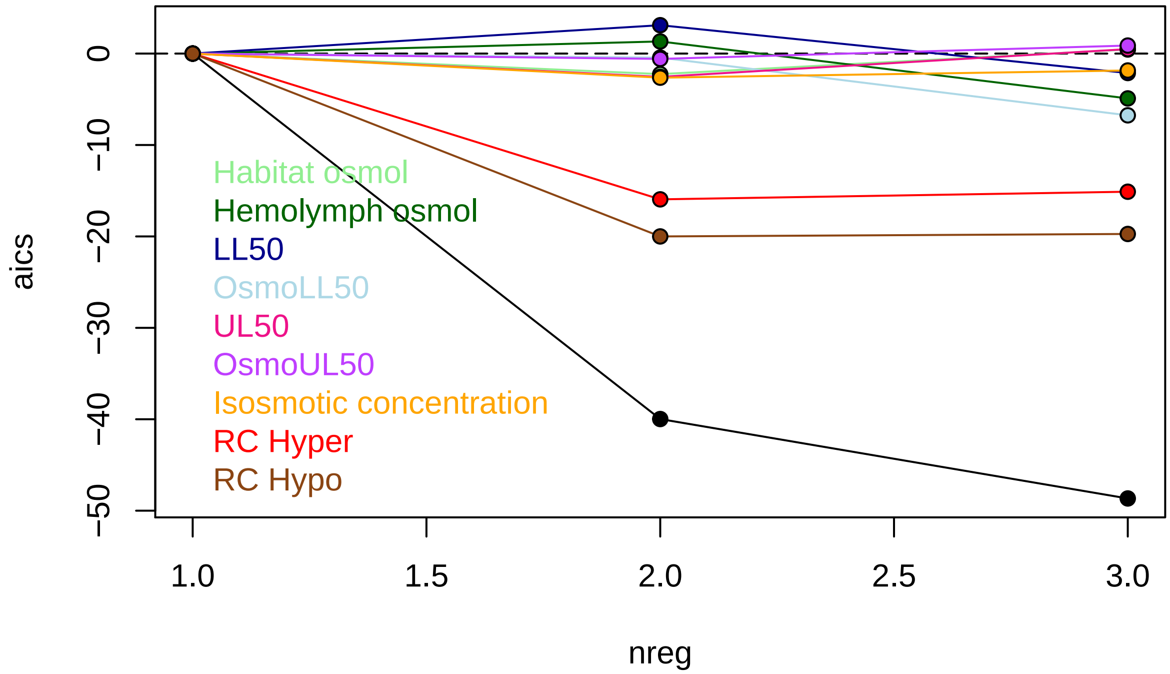

Now let's alter the dAIC threshold criterion and see if that changes the result.

```
fwd1<-runSurface(uca.phylog, uca.data, aic_threshold = - 2)
rsum1<-surfaceSummary(fwd1$bwd)
```

```r
newkk1<-length(fwd1$bwd)
surfaceTreePlot(uca.phylog, fwd1$bwd[[newkk1]], cols = c("blue", "red", "black"))
```

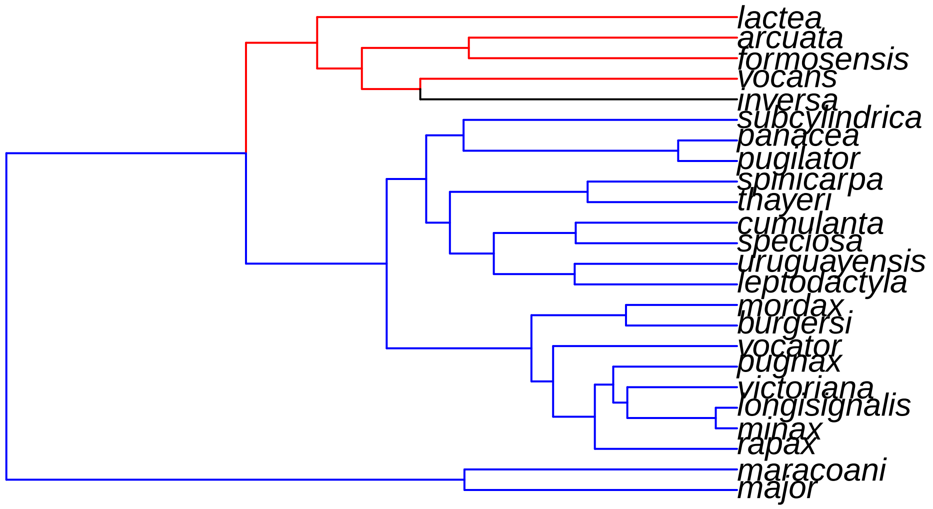

Changing the dAIC threshold criteria **did not change** the results.

Ancestral state estimation using ML

We'll estimate the ancestral states of each character at each node of the tree using the Maximum Likelihood method for continuous characters. This method has the advantage of calculating the 95% CI around each estimate, as a way of tackling uncertainty.

Now, let's map the traits onto the tree:

```r
aa<-contMap(uca.phylog, OsmoHem, plot = FALSE)
ab<-setMap(aa, colors=c("blue","green", "yellow", "red"))
plot(ab)
```

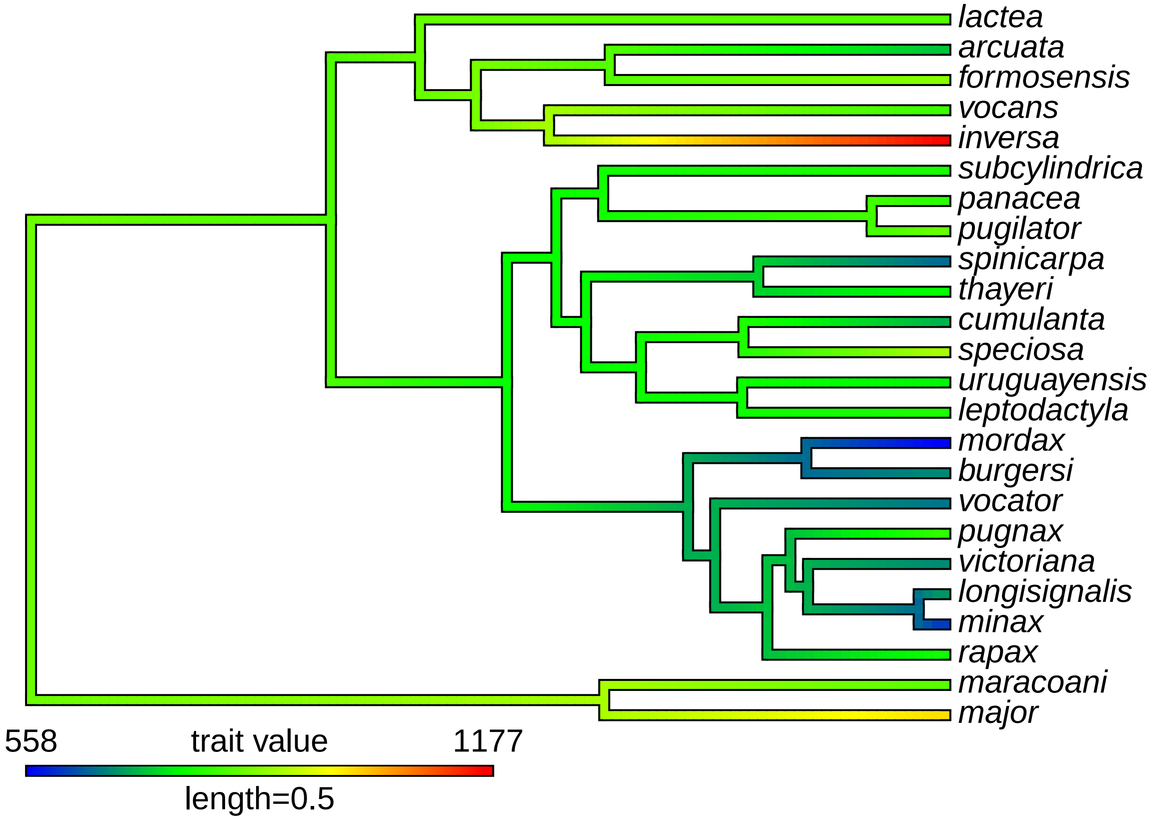


```
az<-contMap(uca.phylog, Habitat, plot = FALSE)
az<-setMap(az, colors=c("blue","green", "yellow", "red"))
plot(az)
```

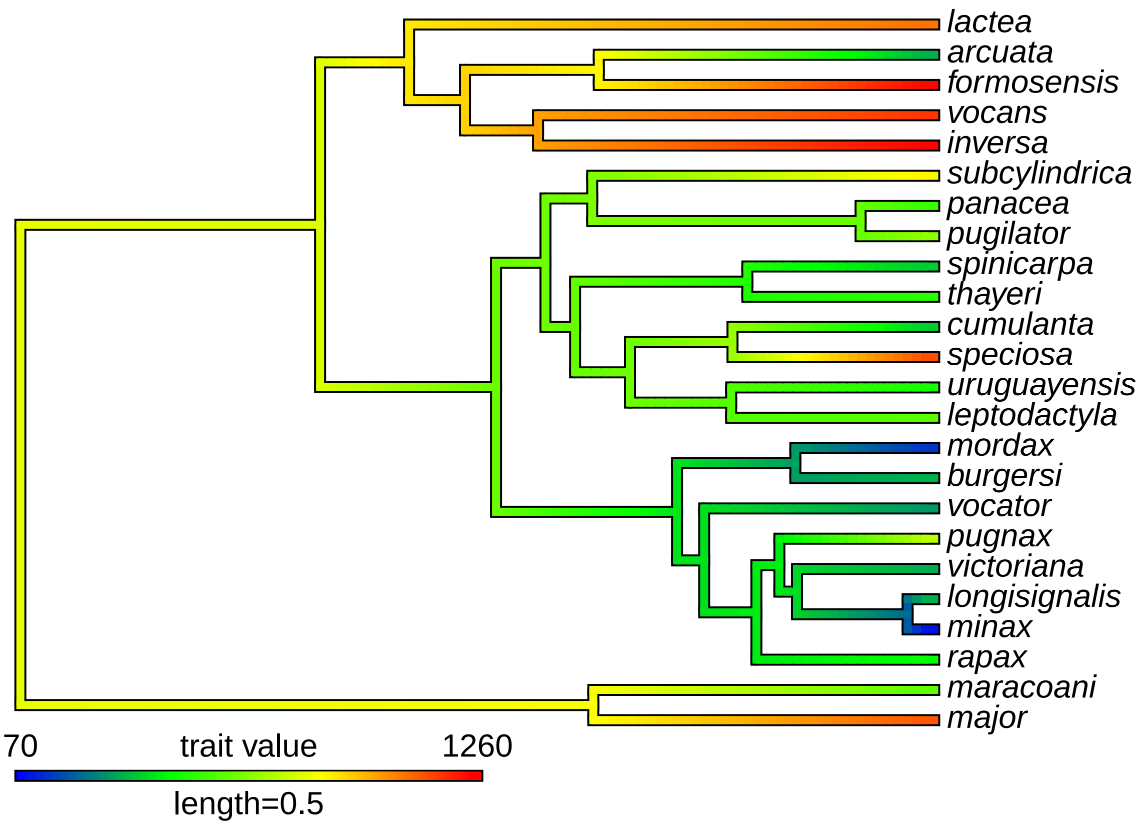


```
ax<-contMap(uca.phylog, PI, plot = FALSE)
az<-setMap(ax, colors=c("blue","green", "yellow", "red"))
plot(ax)
```

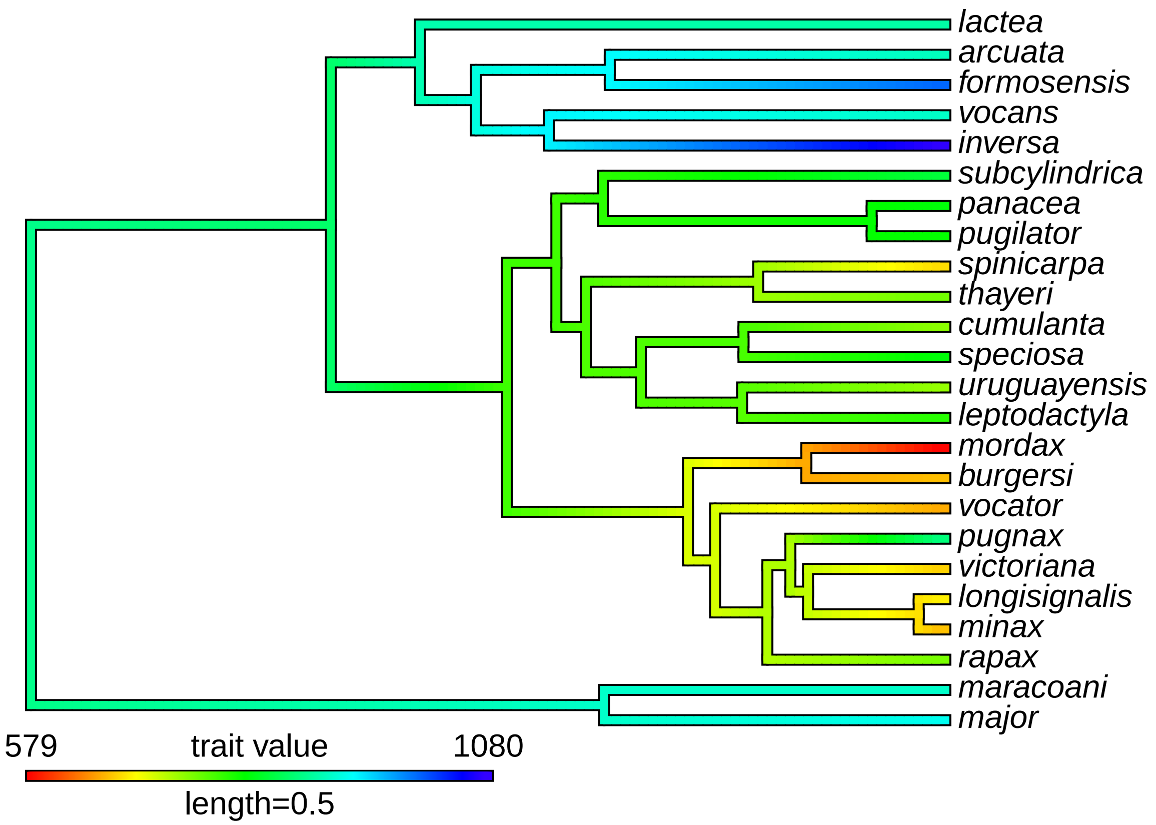


```
ac<-contMap(uca.phylog, Hypo, plot = FALSE)
ac<-setMap(ac, colors=c("blue","green", "yellow", "red"))
plot(ac)
```

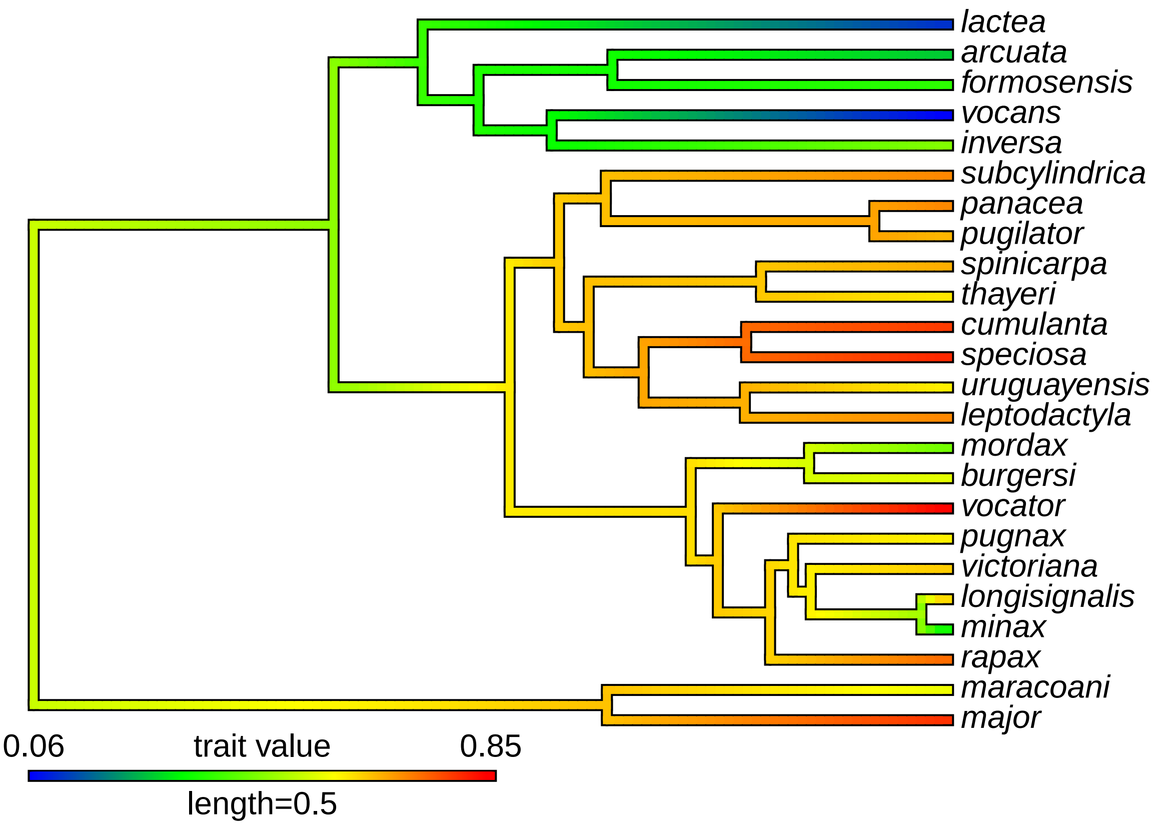


```
ad<-contMap(uca.phylog, Hyper, plot = FALSE)
ad<-setMap(az, colors=c("blue","green", "yellow", "red"))
plot(ad)
```

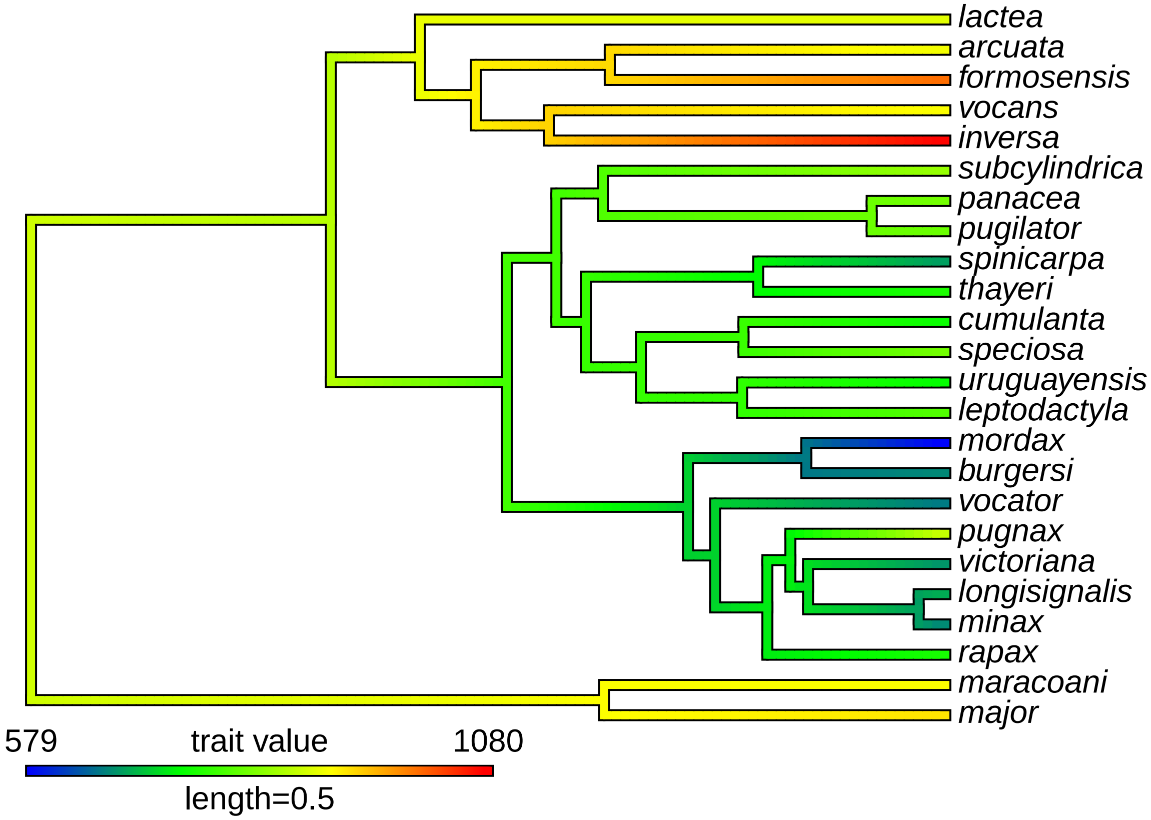

Alternatively, we can plot the ACE of all traits in a scattergram form, along with a traitgram, all teghether.

```
fancyTree(uca.phylog, X=as.matrix(uca.data[,c(1,2,7,8,9)]), type = "scattergram")
```

```
## Computing multidimensional phylogenetic scatterplot matrix...
```

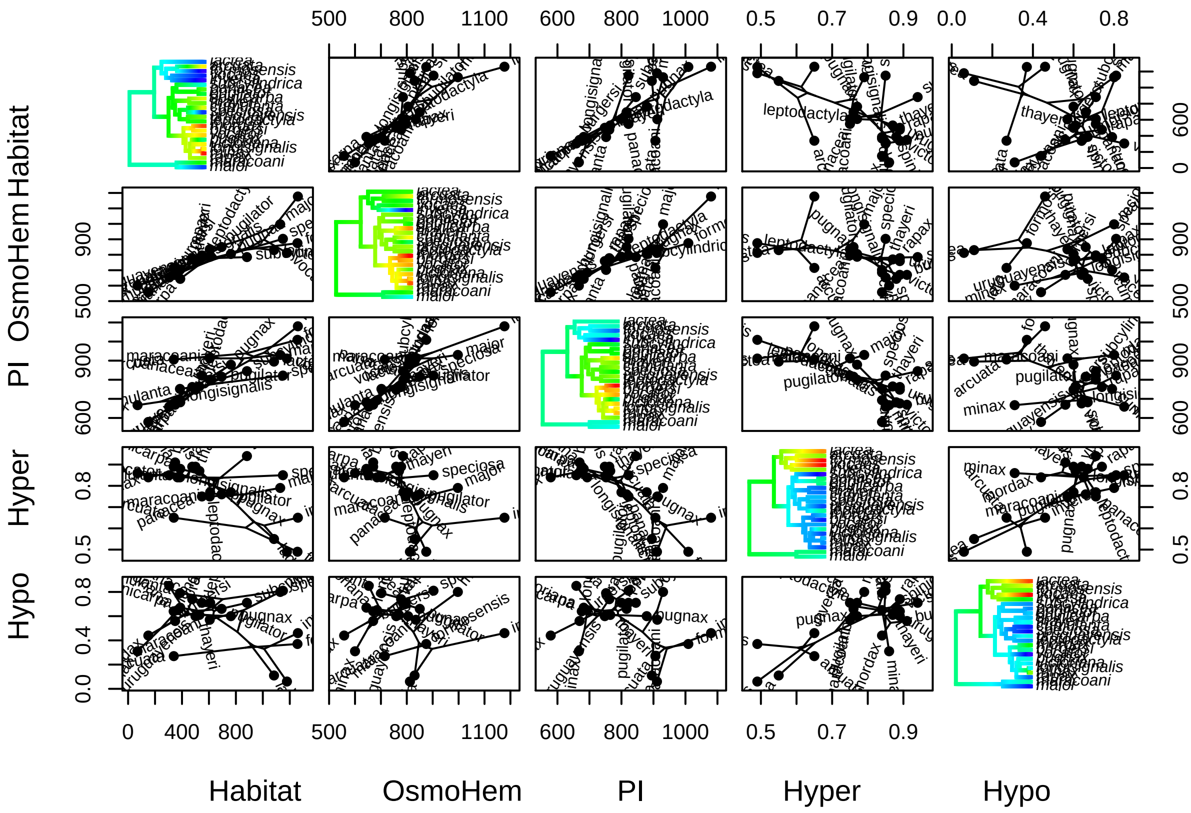

We can also easily compare pairs of traits by plotting phylogenies with estimated ancestral states face-to-face, as follows:

```
uca.phylog<-untangle(uca.phylog,method="read.tree")
layout(matrix(1:3,1,3),widths=c(0.44,0.12,0.44))
par(cex=0.8)
contMap(uca.phylog, OsmoHem, ftype = "off", sig = 1, legend = 3)
ylim<-c(1-0.12*(length(uca.phylog$tip.label)-1),length(uca.phylog$tip.label))
plot.new(); plot.window(xlim=c(-0.1,0.1),ylim=ylim)
text(rep(0,length(uca.phylog$tip.label)), 1:length(uca.phylog$tip.label),uca.phylog$tip.label)
contMap(uca.phylog, Habitat, ftype="off",direction="leftwards", sig=1,legend=3)
```

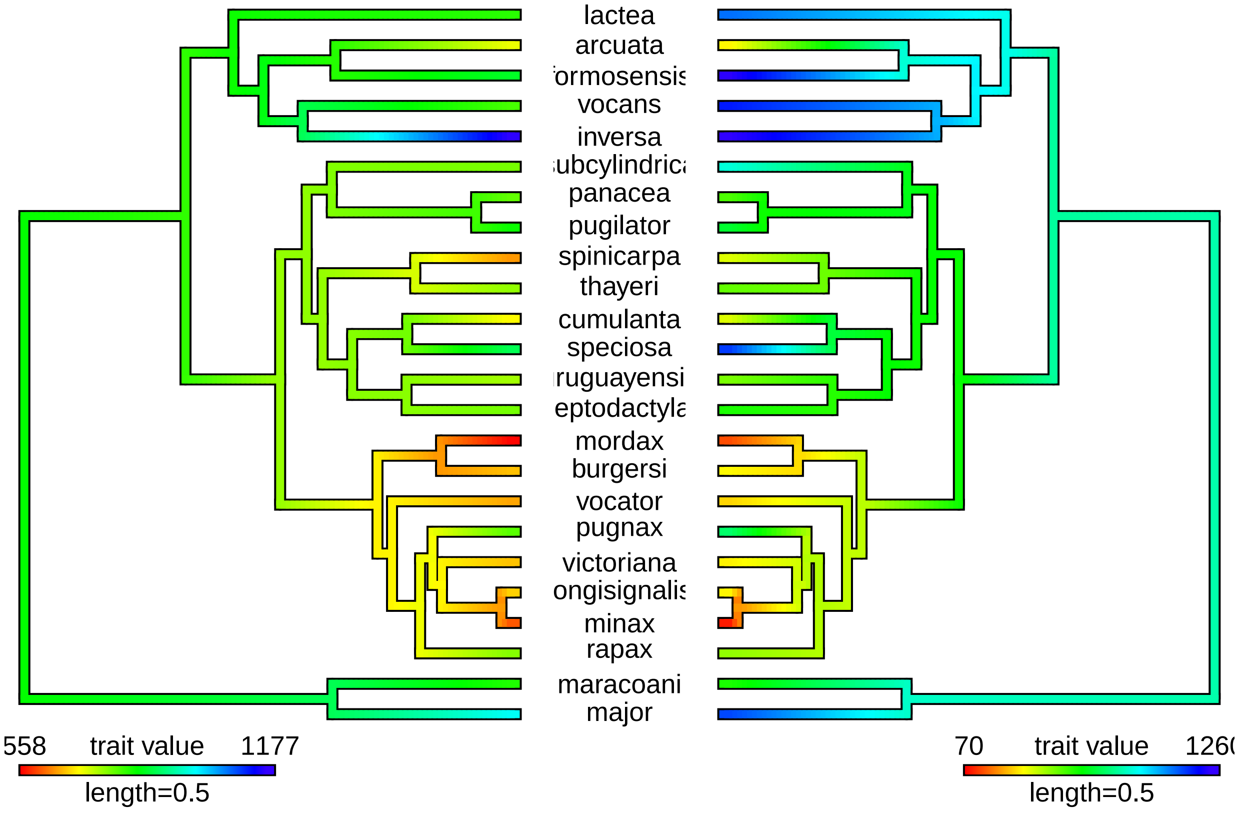

Figure 4. Hemolymph osmolality (left) and Habitat osmolality (right)

```
layout(matrix(1:3,1,3),widths=c(0.44,0.12,0.44))
par(cex=1)
contMap(uca.phylog, PI, ftype = "off", sig = 1, legend = 3)
ylim<-c(1-0.12*(length(uca.phylog$tip.label)-1),length(uca.phylog$tip.label))
plot.new(); plot.window(xlim=c(-0.1,0.1),ylim=ylim)
text(rep(0,length(uca.phylog$tip.label)), 1:length(uca.phylog$tip.label),uca.phylog$tip.label)
contMap(uca.phylog, Habitat, ftype="off",direction="leftwards", sig=1,legend=3)
```

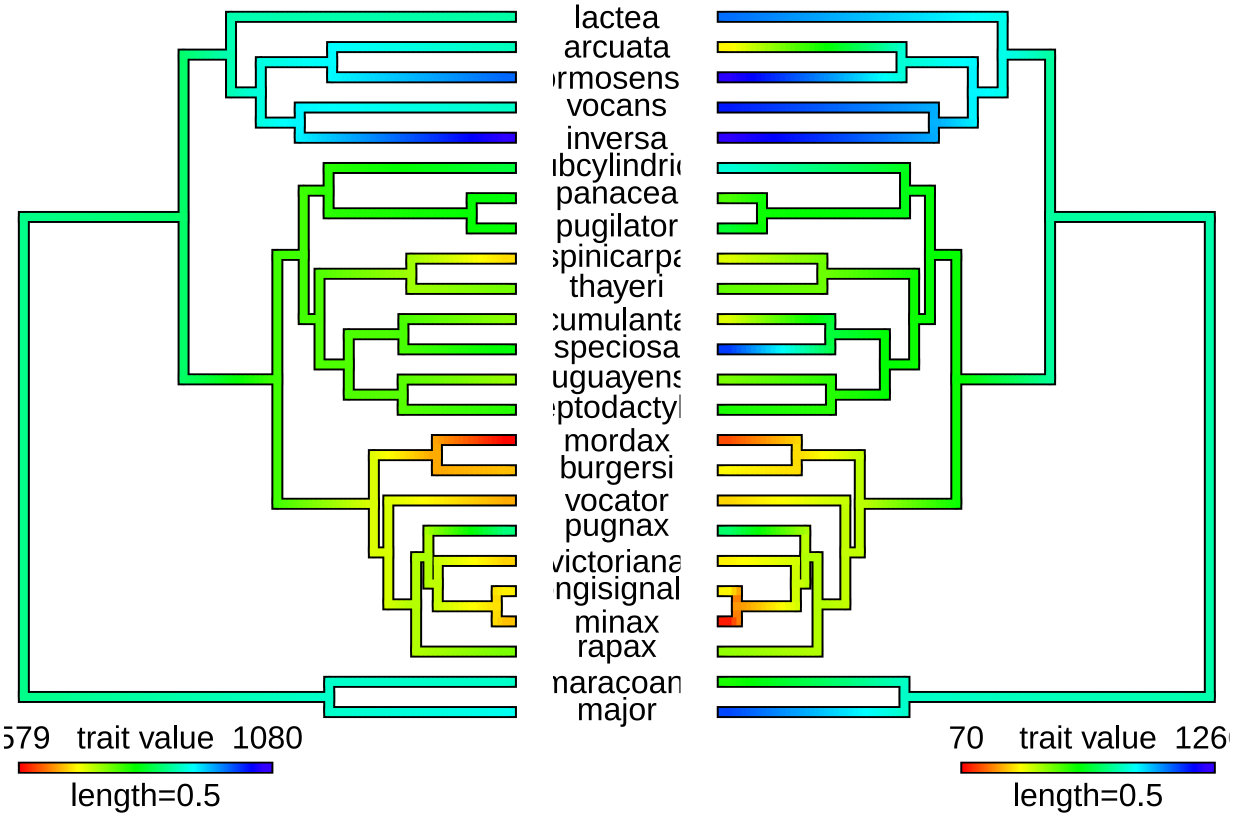

Figure 5. Isosmotic concentration (left) and Habitat osmolality (right)

```
layout(matrix(1:3,1,3),widths=c(0.44,0.12,0.44))
par(cex=1.2)
contMap(uca.phylog, Hyper, ftype = "off", sig = 1, legend = 3)
ylim<-c(1-0.12*(length(uca.phylog$tip.label)-1),length(uca.phylog$tip.label))
plot.new(); plot.window(xlim=c(-0.1,0.1),ylim=ylim)
text(rep(0,length(uca.phylog$tip.label)), 1:length(uca.phylog$tip.label),uca.phylog$tip.label)
contMap(uca.phylog, Hypo, ftype="off",direction="leftwards", sig=1,legend=3)
```

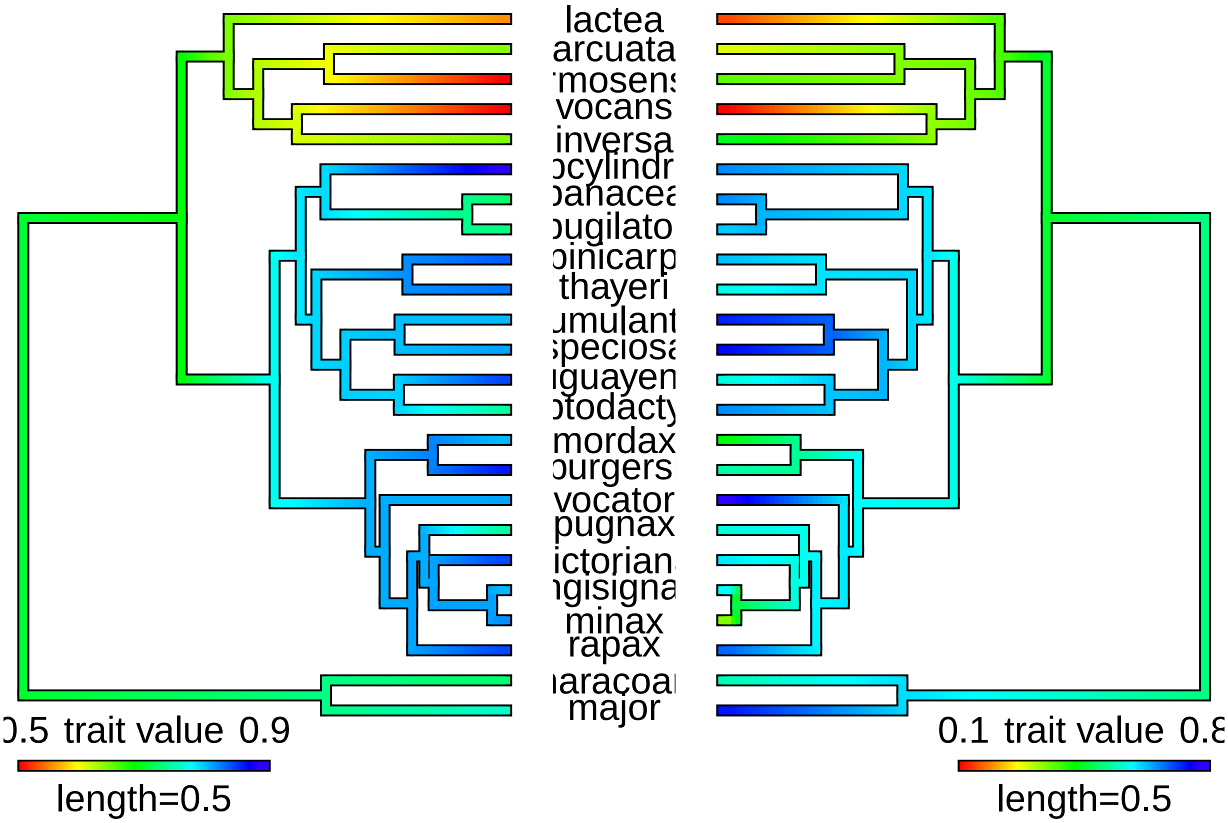

Figure 6. Hyper-regulatry index (left) and Hyporegulatory index (right)

ACE with BM

Now let's estimate the ancestral character state using BM as a model:

```
OH_ace<-fastAnc(uca.phylog, OsmoHem, CI=TRUE)
HA_ace<-fastAnc(uca.phylog, Habitat, CI=TRUE)
PI_ace<-fastAnc(uca.phylog, PI, CI=TRUE)
Hye_ace<-fastAnc(uca.phylog, Hyper, CI=TRUE)
Hyo_ace<-fastAnc(uca.phylog, Hypo, CI=TRUE)
```

We can also use a more "traditional" plot, showing the estimated ancestral values at the nodes

```
plot.phylo(uca.phylog)
nodelabels(round(OH_ace$ace, digits=1))
```

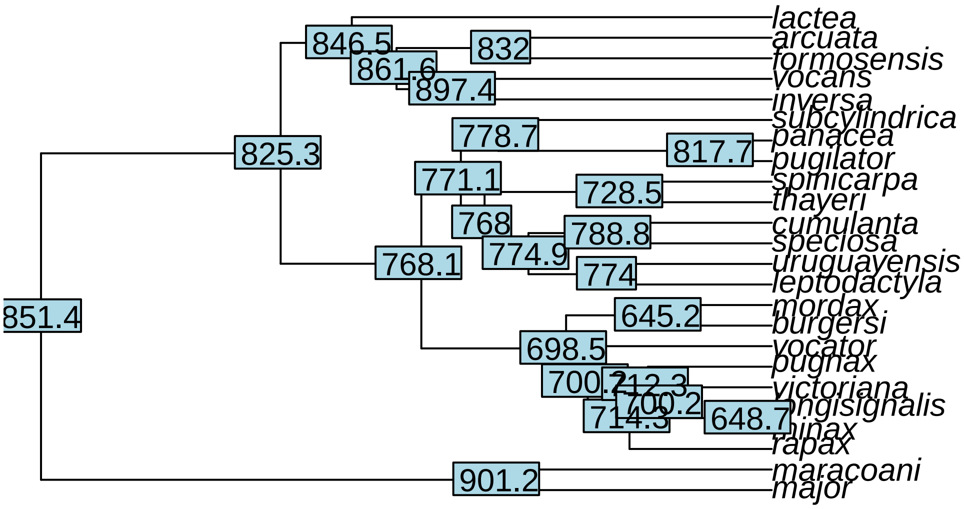


```
plot.phylo(uca.phylog)
nodelabels(round(HA_ace$ace, digits=1))
```

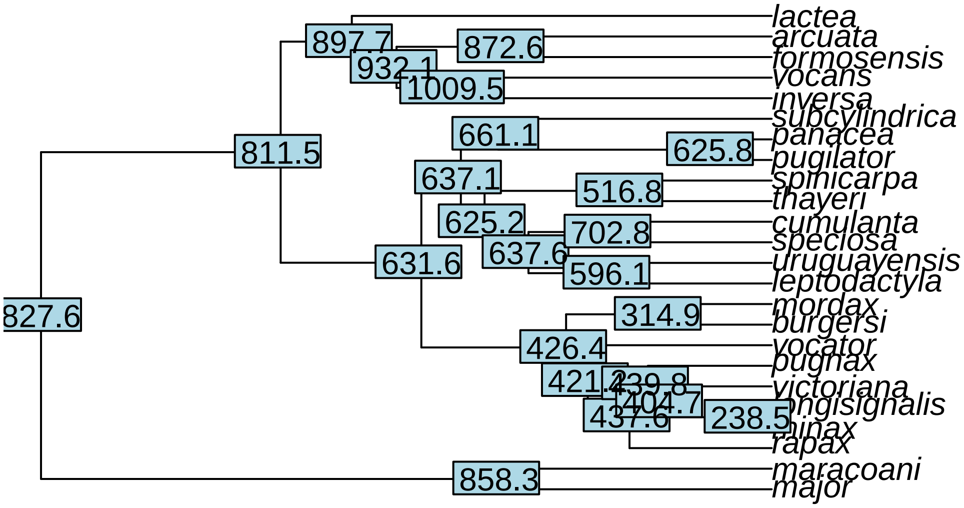


```
plot.phylo(uca.phylog)
nodelabels(round(PI_ace$ace, digits=1))
```

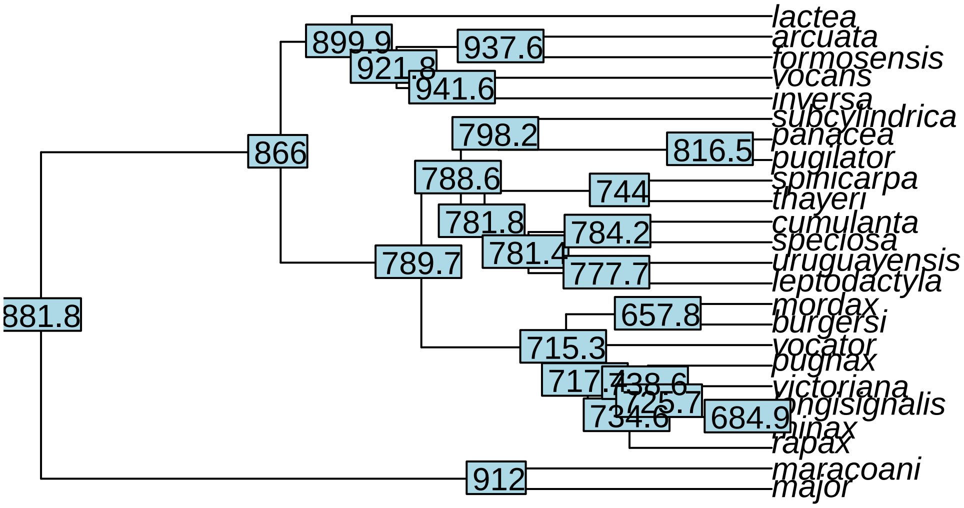

```r
plot.phylo(uca.phylog)
nodelabels(round(Hyo_ace$ace, digits=1))
```

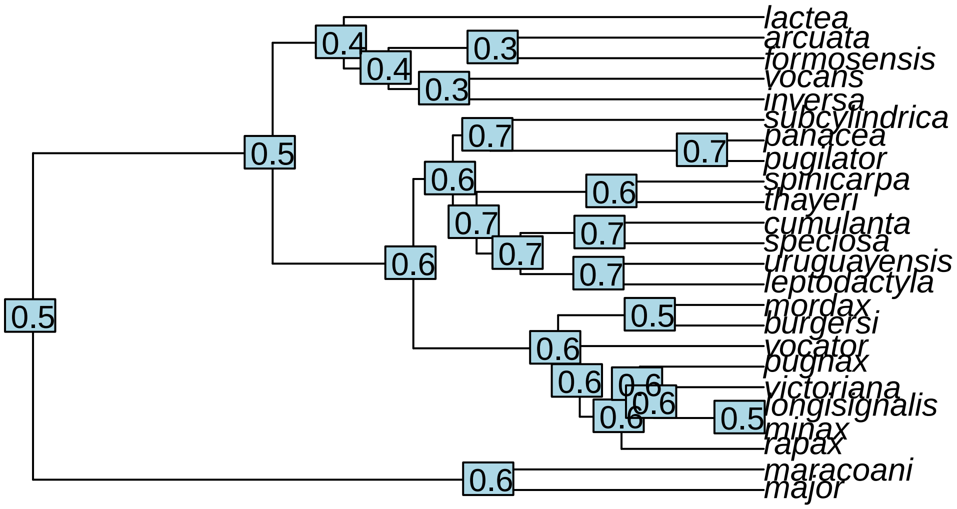


```r
plot.phylo(uca.phylog)
nodelabels(round(Hye_ace$ace, digits=1))
```

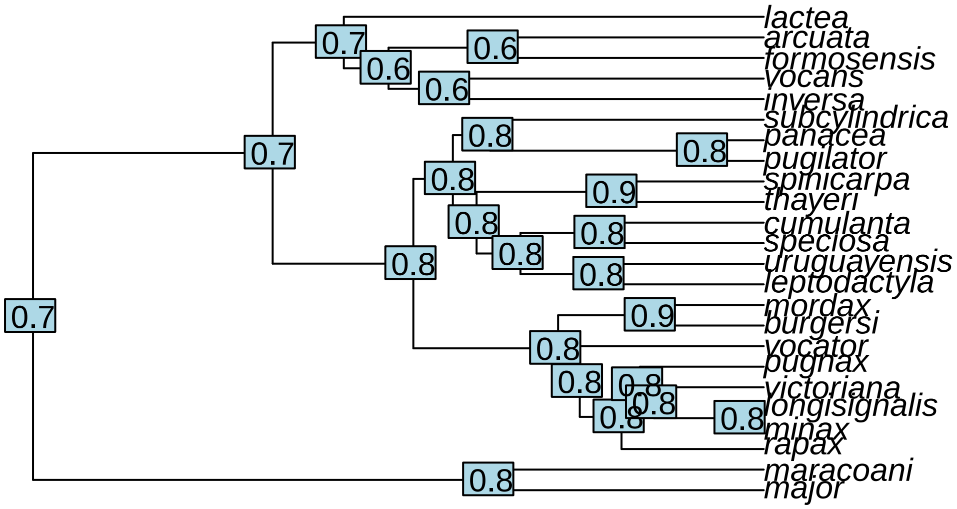

Now let's show the CI for each node for each trait

```r
plot.phylo(uca.phylog)
nodelabels()#to check the node numbers in the tree
```

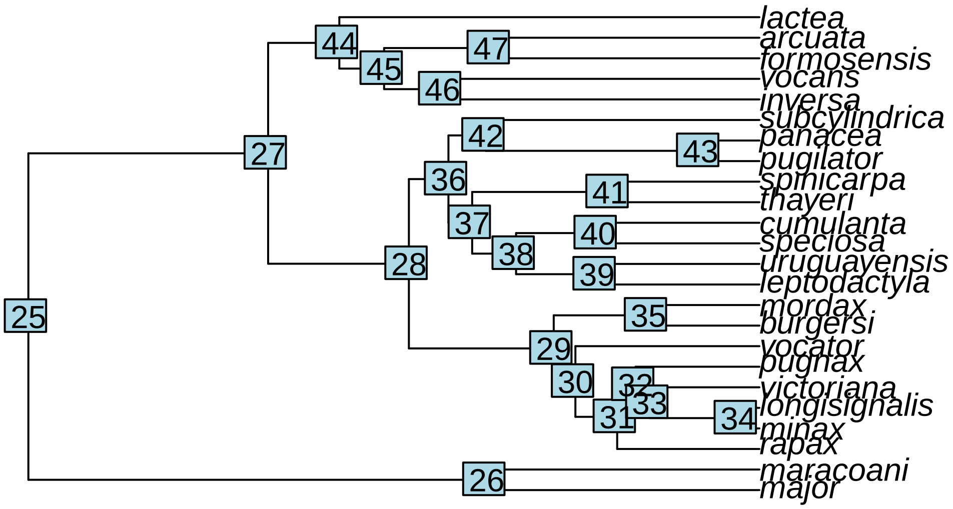


```
OH_ace$CI95
```

```
##          [,1]      [,2]
## 25 646.6109 1056.1250
## 26 750.6955 1051.6609
## 27 694.4630  956.1611
## 28 664.7612  871.4855
## 29 607.9300  788.9904
## 30 614.1281  786.3199
## 31 636.7214  791.9518
## 32 638.3683  786.2754
## 33 625.7564  774.6919
## 34 604.9639  692.4420
## 35 556.1987  734.2938
## 36 677.3239  864.8382
## 37 674.5368  861.4826
## 38 678.8916  870.9670
## 39 672.9780  874.9551
## 40 687.9274  889.7130
## 41 625.9609  830.9648
## 42 674.2110  883.2261
## 43 745.5691  889.7466
## 44 723.5981  969.4062
## 45 743.0366  980.1035
## 46 771.6749 1023.0899
## 47 705.8339  958.2337
```

```
HA_ace$CI95
```

```
##           [,1]      [,2]
## 25 235.84016 1419.2695
## 26 423.46886 1293.2102
## 27 433.31863 1189.5838
## 28 332.88972  930.2896
## 29 164.76862  688.0038
## 30 172.39350  669.9998
## 31 213.27675  661.8677
## 32 226.10251  653.5301
```

```
## 33 189.53929  619.9388
## 34 112.11422  364.9118
## 35  57.60655  572.2724
## 36 366.15686  908.0431
## 37 355.03690  895.2800
## 38 360.05613  915.1230
## 39 304.28577  887.9666
## 40 411.22638  994.3541
## 41 220.59121  813.0194
## 42 359.12050  963.1401
## 43 417.51657  834.1662
## 44 542.48035 1252.8260
## 45 589.52392 1274.6089
## 46 646.22884 1372.7775
## 47 507.88559 1237.2801
```

```
Hye_ace$CI95
```

```
##          [,1]      [,2]
## 25 0.5871387 0.8638716
## 26 0.6581061 0.8614862
## 27 0.6191449 0.7959898
## 28 0.7351549 0.8748507
## 29 0.7837137 0.9060669
## 30 0.7883362 0.9046964
## 31 0.7964074 0.9013058
## 32 0.7945937 0.8945433
## 33 0.7994481 0.9000927
## 34 0.8204183 0.8795324
## 35 0.8036862 0.9240355
## 36 0.7581161 0.8848306
## 37 0.7652329 0.8915633
## 38 0.7666899 0.8964866
## 39 0.7625518 0.8990396
## 40 0.7701662 0.9065247
## 41 0.7893391 0.9278724
## 42 0.7555158 0.8967596
## 43 0.7148675 0.8122968
## 44 0.5698967 0.7360038
## 45 0.5495038 0.7097040
## 46 0.5285982 0.6984941
## 47 0.5178777 0.6884391
```

```
Hyo_ace$CI95
```

```
##           [,1]      [,2]
## 25 0.12643824 0.9359832
## 26 0.35225146 0.9472129
## 27 0.21054357 0.7278796
## 28 0.40189014 0.8105517
## 29 0.44235452 0.8002824
## 30 0.47090420 0.8113003
## 31 0.47835822 0.7852245
## 32 0.46750810 0.7598972
## 33 0.45417892 0.7486010
```

```
## 34 0.40657917 0.5795096
## 35 0.36884910 0.7209150
## 36 0.45522649 0.8259130
## 37 0.46838891 0.8379514
## 38 0.48936411 0.8690671
## 39 0.46749311 0.8667699
## 40 0.54062100 0.9395195
## 41 0.44200289 0.8472635
## 42 0.44660346 0.8597933
## 43 0.53865529 0.8236715
## 44 0.13852589 0.6244499
## 45 0.12096893 0.5896130
## 46 0.08111484 0.5781228
## 47 0.09015543 0.5891102
```

```
PI_ace$CI95
```

```
##         [,1]      [,2]
## 25 730.2889 1033.3536
## 26 800.6492 1023.3814
## 27 769.1905  962.8626
## 28 713.2130  866.2013
## 29 648.3360  782.3314
## 30 653.6687  781.1008
## 31 677.1431  792.0229
## 32 683.8827  793.3428
## 33 670.6080  780.8291
## 34 652.5434  717.2823
## 35 591.8887  723.6896
## 36 719.1931  857.9648
## 37 712.6425  850.9935
## 38 710.2782  852.4254
## 39 702.9456  852.4206
## 40 709.5309  858.8642
## 41 668.1584  819.8734
## 42 720.8248  875.5084
## 43 763.1367  869.8366
## 44 808.9859  990.8985
## 45 834.0512 1009.4947
## 46 848.6046 1034.6666
## 47 844.2292 1031.0200
```

An easy and intuitive way to illustrate uncertainty in the estimated ancestral states is plotting a density phenogram

```
fancyTree(uca.phylog,type="phenogram95",x=OsmoHem,spread.cost=c(1,0))
```

```
## Computing density traitgram...
```

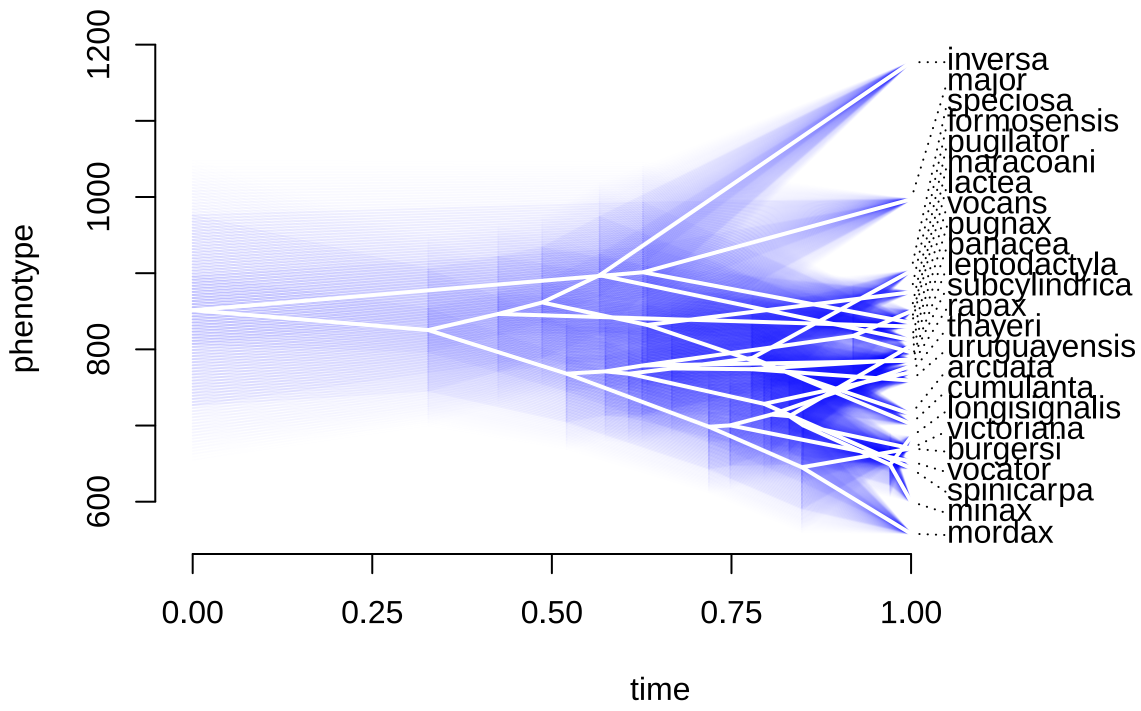


```
fancyTree(uca.phylog,type="phenogram95",x=Habitat,spread.cost=c(1,0))
```

```
## Computing density traitgram...
```

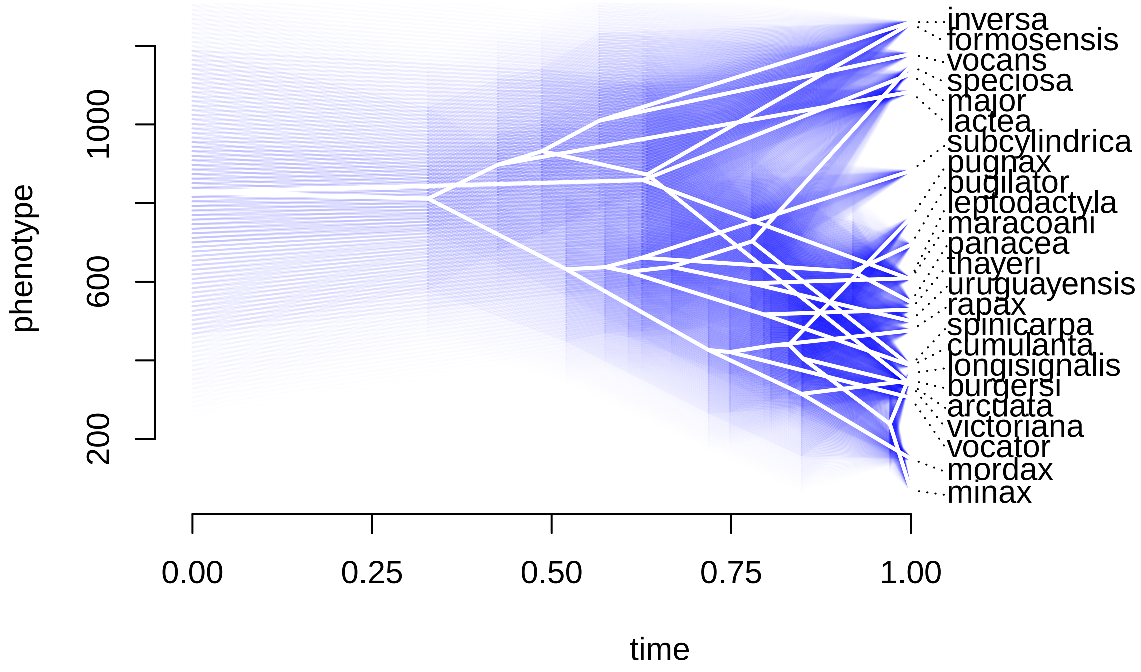


```
fancyTree(uca.phylog,type="phenogram95",x=PI,spread.cost=c(1,0))
```

```
## Computing density traitgram...
```

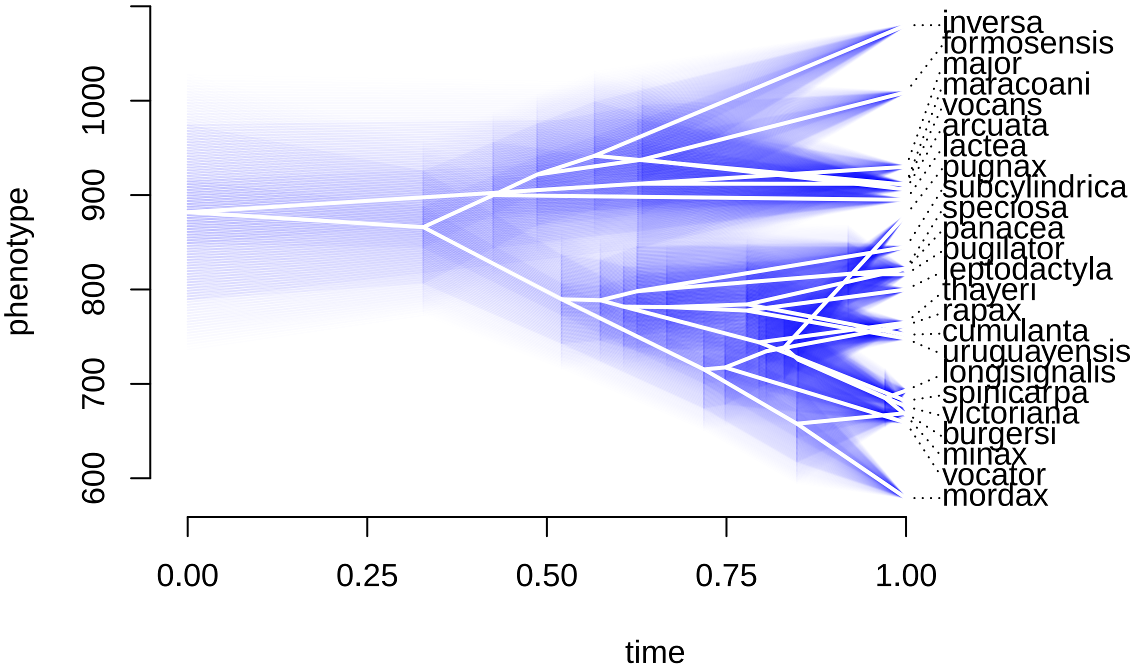


```
fancyTree(uca.phylog,type="phenogram95",x=Hyper,spread.cost=c(1,0))
```

```
## Computing density traitgram...
```

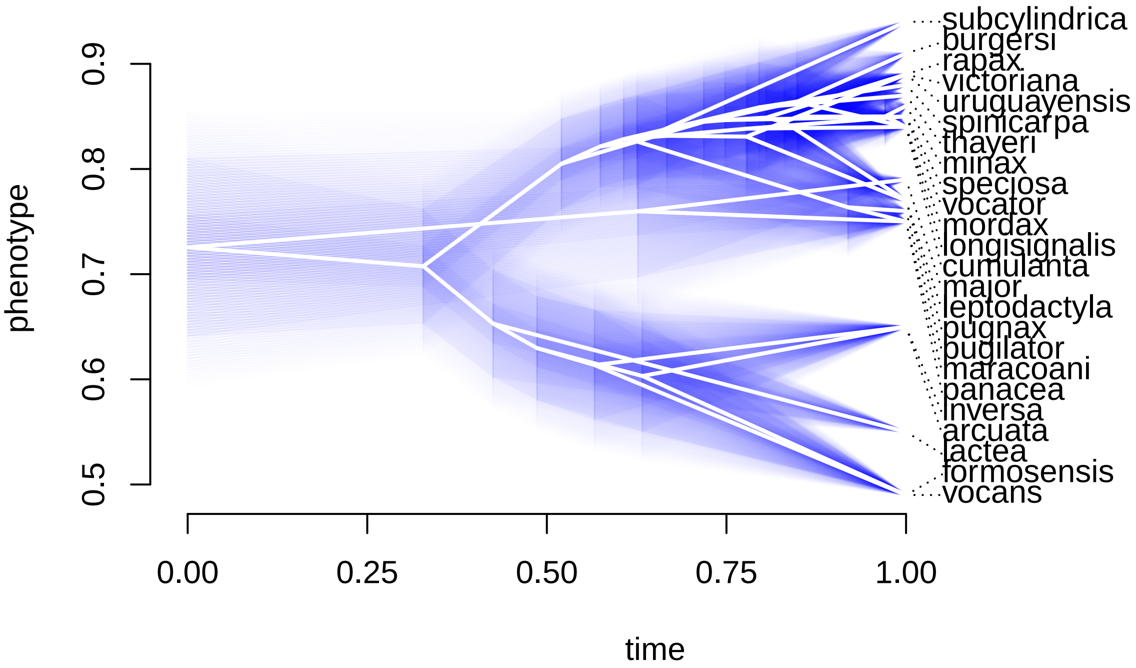


```
fancyTree(uca.phylog,type="phenogram95",x=Hypo,spread.cost=c(1,0))
```

```
## Computing density traitgram...
```

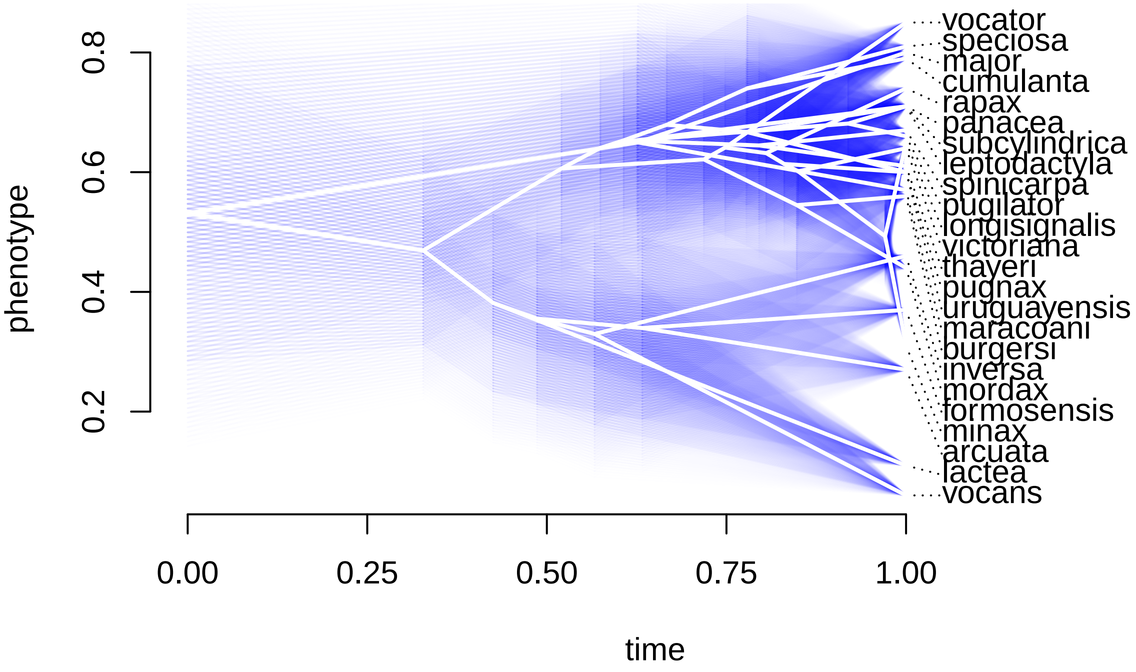

PGLS analyses

We followed Revell (2010) MEE and run the PGLS while estimating the alpha parameter of the OU model at the same time.

```
summary(gls(OsmoHem~Habitat+1,Trait,corPagel(1, phy=uca.phylog),method = "ML"))
```

```
## Generalized least squares fit by maximum likelihood
##   Model: OsmoHem ~ Habitat + 1
##   Data: Trait
##        AIC      BIC    logLik
##   250.6906 255.4028 -121.3453
##
## Correlation Structure: corPagel
##  Formula: ~1
##  Parameter estimate(s):
##   lambda
## 1.030017
##
## Coefficients:
##                Value Std.Error   t-value p-value
## (Intercept) 614.6204  60.13451      10.2       0
## Habitat       0.2862   0.00000 1316897.6       0
##
##  Correlation:
##         (Intr)
## Habitat 0
##
## Standardized residuals:
##         Min          Q1         Med          Q3         Max
## -1.33418341 -0.54328717 -0.29838801  0.08691178  1.92226419
```

```
##
## Residual standard error: 104.9666
## Degrees of freedom: 24 total; 22 residual
```

```r
summary(gls(Hyper~Habitat+1,Trait,corPagel(1,phy=uca.phylog),method = "ML"))
```

```
## Generalized least squares fit by maximum likelihood
##   Model: Hyper ~ Habitat + 1
##   Data: Trait
##         AIC       BIC   logLik
##   -64.35733 -59.64512 36.17867
##
## Correlation Structure: corPagel
##  Formula: ~1
##  Parameter estimate(s):
##   lambda
## 1.030017
##
## Coefficients:
##                  Value  Std.Error    t-value p-value
## (Intercept)  0.7809414 0.07004959    11.1484       0
## Habitat     -0.0000673 0.00000003 -2675.6583       0
##
##  Correlation:
##         (Intr)
## Habitat 0
##
## Standardized residuals:
##         Min          Q1         Med          Q3         Max
## -1.73011527  0.07270759  0.68512213  1.03697137  1.78658718
##
## Residual standard error: 0.1222736
## Degrees of freedom: 24 total; 22 residual
```

```r
summary(gls(PI~Habitat+1,Trait,corPagel(1, phy=uca.phylog),method = "ML"))
```

```
## Generalized least squares fit by maximum likelihood
##   Model: PI ~ Habitat + 1
##   Data: Trait
##        AIC      BIC    logLik
##   273.4356 278.1478 -132.7178
##
## Correlation Structure: corPagel
##  Formula: ~1
##  Parameter estimate(s):
##    lambda
## 0.9147465
##
## Coefficients:
##                Value Std.Error   t-value p-value
## (Intercept) 711.7080  57.34367 12.411273       0
## Habitat       0.2045   0.03970  5.150309       0
##
##  Correlation:
##         (Intr)
```

```
## Habitat -0.569
##
## Standardized residuals:
##        Min         Q1        Med         Q3        Max
## -1.9062544 -1.1261937 -0.5183342 -0.1152456  1.4580739
##
## Residual standard error: 85.70832
## Degrees of freedom: 24 total; 22 residual
```

```
summary(gls(Hyper~Hypo+1,Trait,corPagel(1,phy=uca.phylog),method = "ML"))
```

```
## Generalized least squares fit by maximum likelihood
##   Model: Hyper ~ Hypo + 1
##   Data: Trait
##         AIC       BIC   logLik
##   -45.53224 -40.82003 26.76612
##
## Correlation Structure: corPagel
##  Formula: ~1
##  Parameter estimate(s):
##    lambda
## 0.9189327
##
## Coefficients:
##                 Value  Std.Error  t-value p-value
## (Intercept) 0.6375819 0.07758582 8.217763  0.0000
## Hypo        0.1677336 0.08844936 1.896380  0.0711
##
##  Correlation:
##      (Intr)
## Hypo -0.607
##
## Standardized residuals:
##         Min          Q1         Med          Q3         Max
## -1.87373697  0.06361261  0.62453881  1.16237659  1.63853153
##
## Residual standard error: 0.1118851
## Degrees of freedom: 24 total; 22 residual
```

```
summary(gls(Hypo~Habitat+1,Trait,corPagel(1,phy=uca.phylog),method = "ML"))
```

```
## Generalized least squares fit by maximum likelihood
##   Model: Hypo ~ Habitat + 1
##   Data: Trait
##         AIC        BIC   logLik
##   -5.570426 -0.8582107 6.785213
##
## Correlation Structure: corPagel
##  Formula: ~1
##  Parameter estimate(s):
##    lambda
## 0.8132011
##
## Coefficients:
##                 Value  Std.Error  t-value p-value
```

```
## (Intercept) 0.4643361 0.15871061 2.925678  0.0078
## Habitat     0.0000854 0.00011989 0.712052  0.4839
##
##  Correlation:
##         (Intr)
## Habitat -0.614
##
## Standardized residuals:
##        Min         Q1        Med         Q3        Max
## -2.1333852 -0.2358656  0.5004599  0.8237106  1.5192996
##
## Residual standard error: 0.2367057
## Degrees of freedom: 24 total; 22 residual
```

Now, let's plot the data for each model.

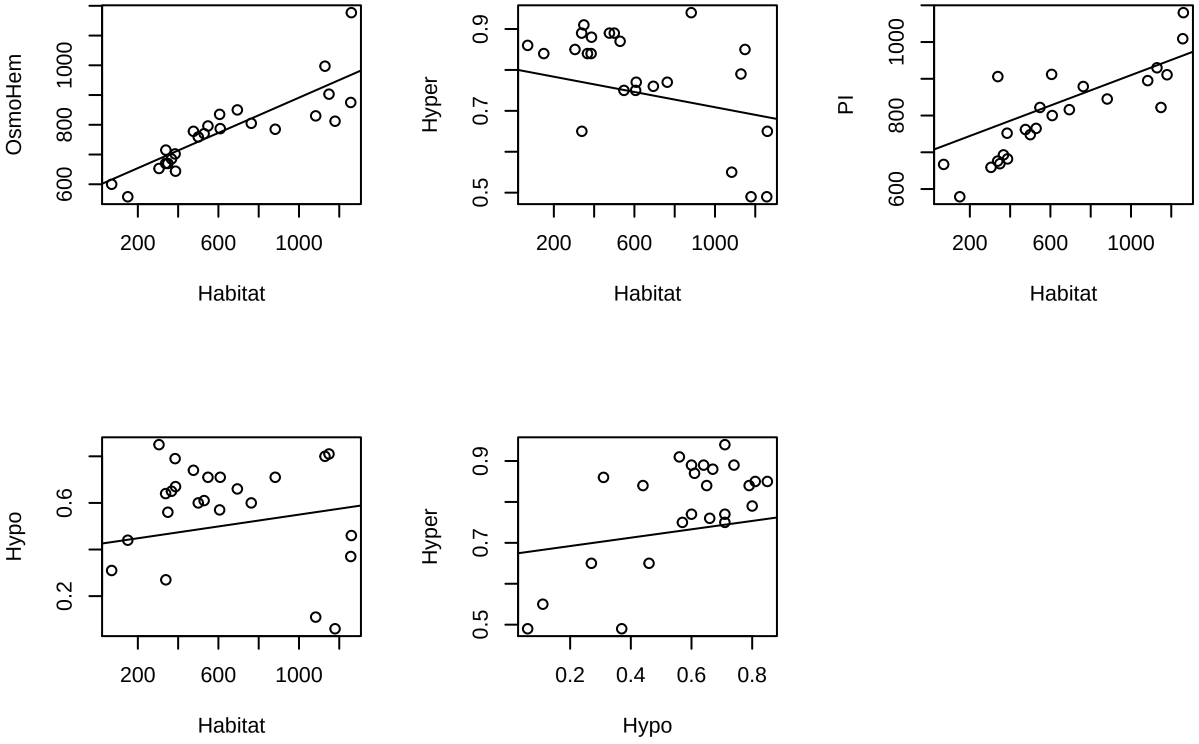

Diagnostics of PGLS

We can use diagnostic plots to check if each PGLS model had problems fitting the data.

```r
par(mfrow = c(1,1))
source("http://ib.berkeley.edu/courses/ib200b/scripts/diagnostics_v3.R")
diagnostics(uca.data[,1],uca.phylog)#for Habitat osmolality
```

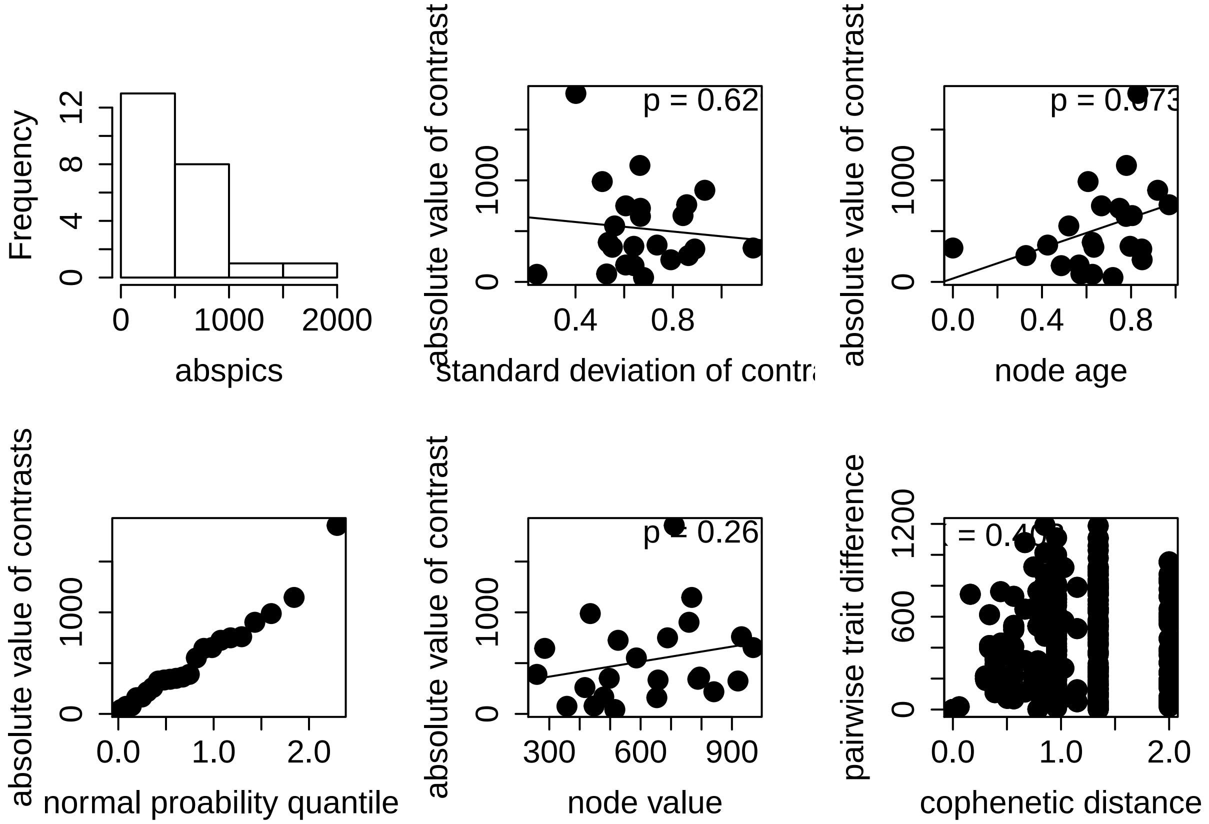


```r
diagnostics(uca.data[,2],uca.phylog)#for hemolymph osmolality
```

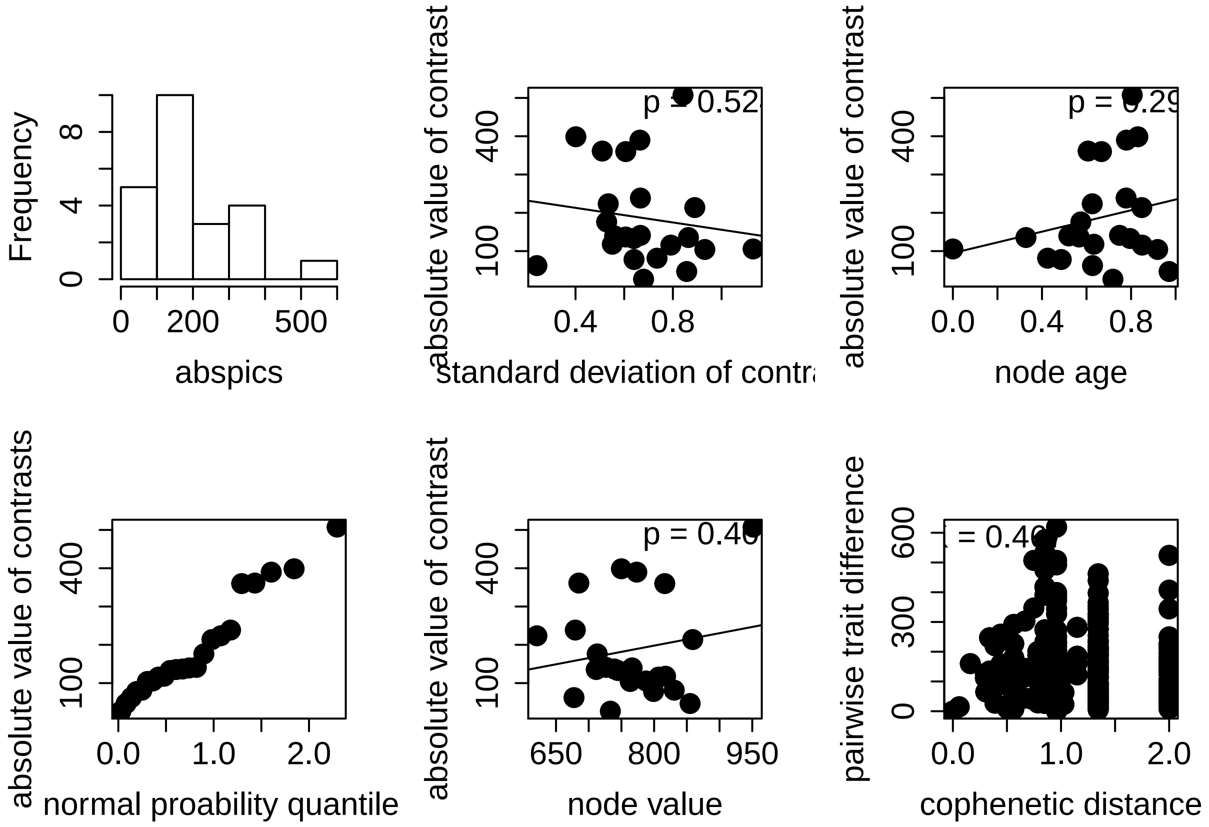

```r
diagnostics(uca.data[,7],uca.phylog)#for isosmotic point
```

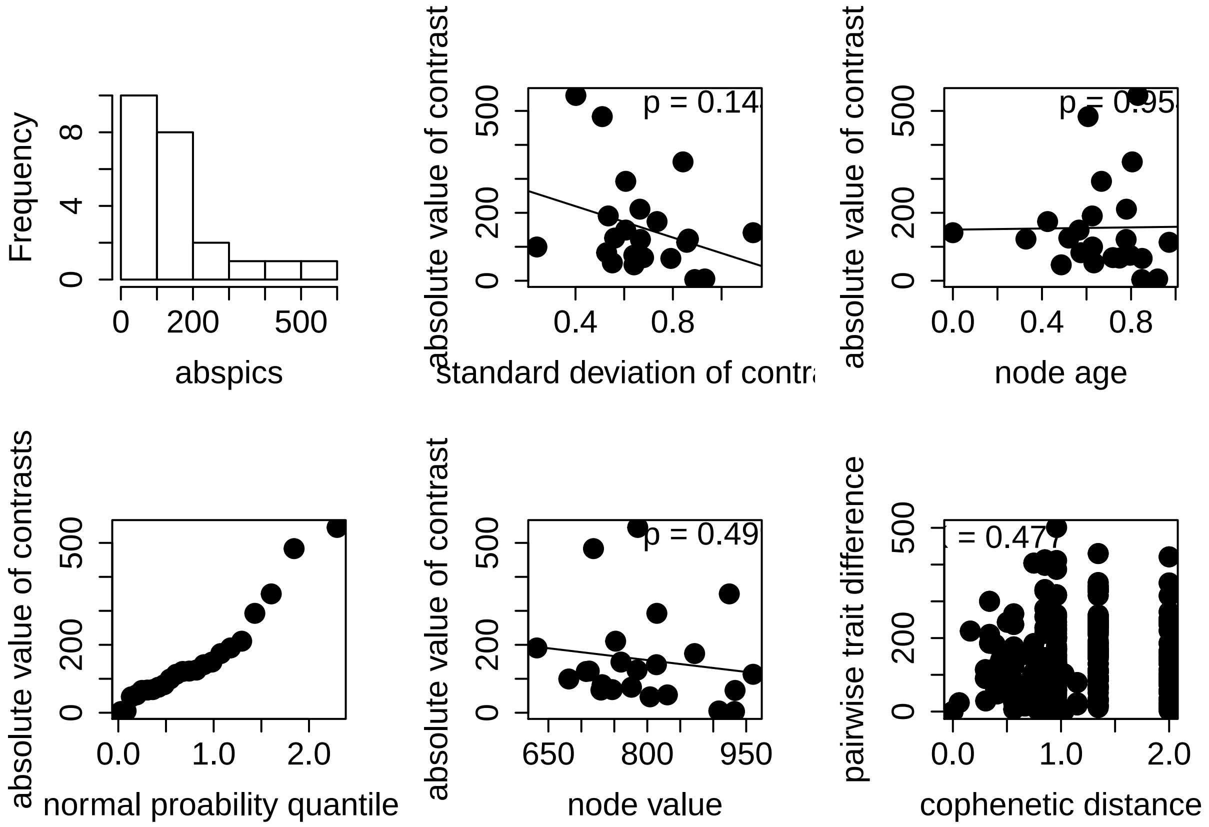


```r
diagnostics(uca.data[,8],uca.phylog)#for hyper-regulatory index
```

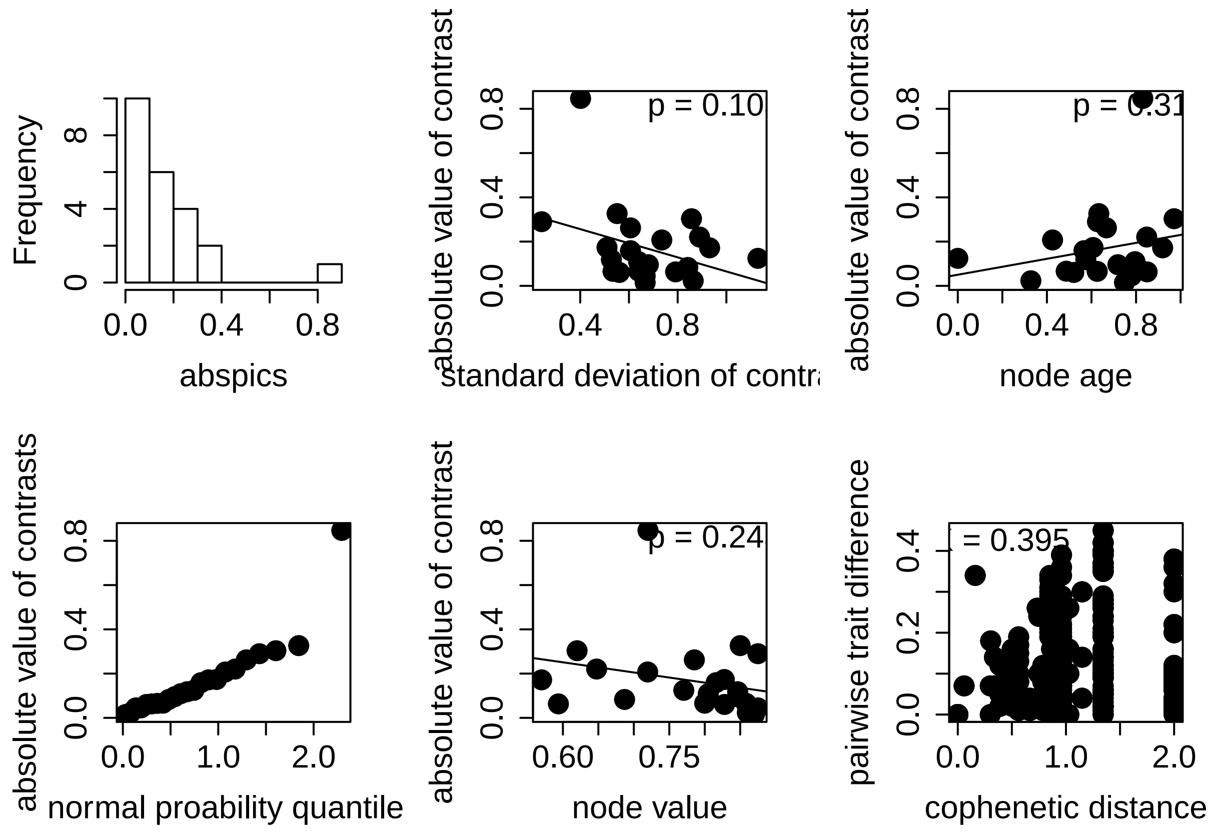


```r
diagnostics(uca.data[,9],uca.phylog)#for hypo-regulatory index
```

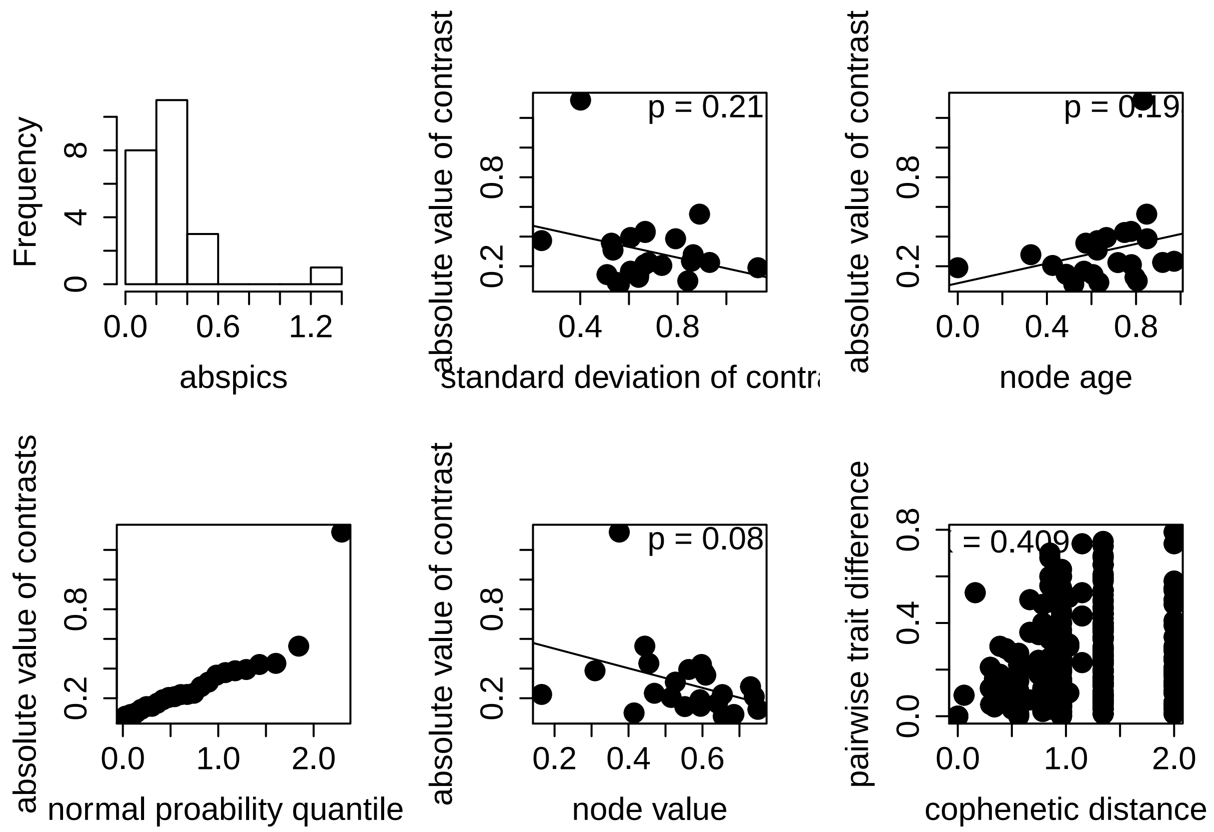

Supplement: S1 File — This file describes all comparative analyses performed in the investigation. (PDF) [file pone.0171870.s001.pdf]
